# Supplementary material for: Highly specific monoclonal antibodies and epitope identification against SARS-CoV-2 nucleocapsid protein for antigen detection tests
Source: Cell Rep Med. 2021 May 16;2(6):100311. doi: 10.1016/j.xcrm.2021.100311 (PMC8126173; doi:10.1016/j.xcrm.2021.100311)
Supplement: Document S1. Figures S1–S8 [file mmc1.pdf]

Cell Reports Medicine, Volume *2*

Supplemental information

Highly specific monoclonal antibodies and epitope identification against SARS-CoV-2 nucleocapsid protein for antigen detection tests

Yutaro Yamaoka, Kei Miyakawa, Sundararaj Stanleyraj Jeremiah, Rikako Funabashi, Koji Okudela, Sayaka Kikuchi, Junichi Katada, Atsuhiko Wada, Toshiki Takei, Mayuko Nishi, Kohei Shimizu, Hiroki Ozawa, Shuzo Usuku, Chiharu Kawakami, Nobuko Tanaka, Takeshi Morita, Hiroyuki Hayashi, Hideaki Mitsui, Keita Suzuki, Daisuke Aizawa, Yukihiro Yoshimura, Tomoyuki Miyazaki, Etsuko Yamazaki, Tadaki Suzuki, Hirokazu Kimura, Hideaki Shimizu, Nobuhiko Okabe, Hideki Hasegawa, and Akihide Ryo

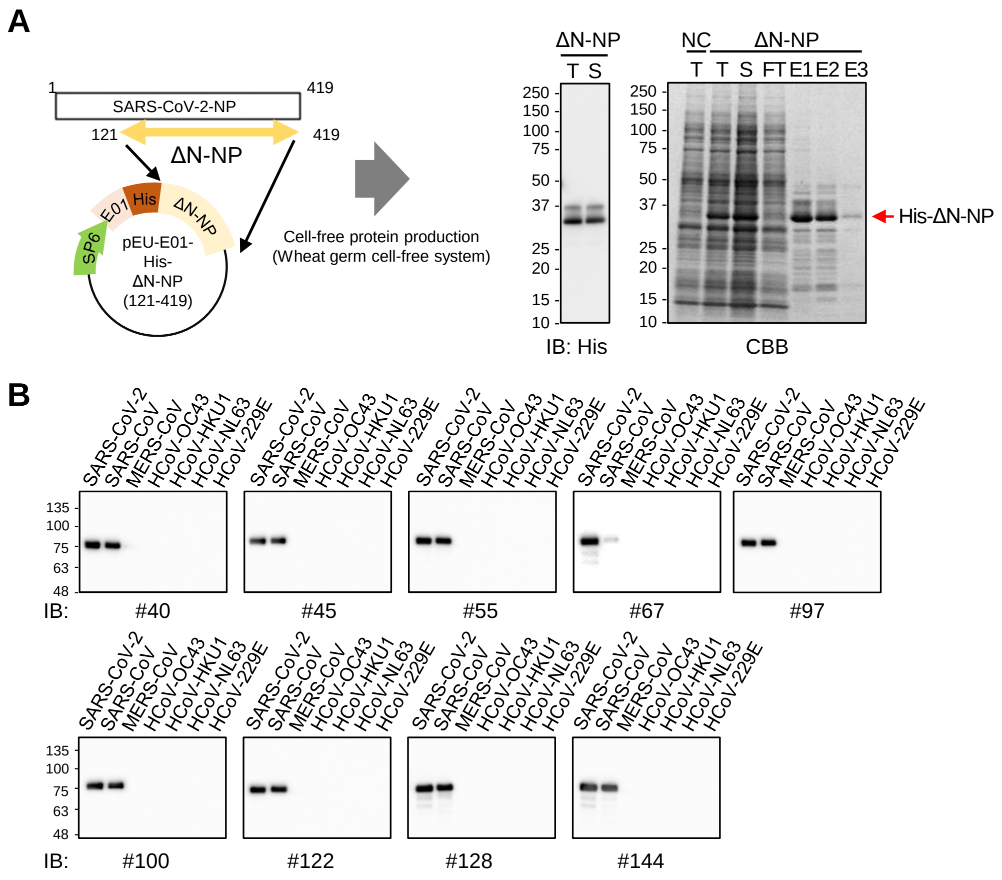

Supplementary Figure 1. (Related to Figure 1) Antigen protein production and specificity screening of developed mAbs.

(A) Schematic representation of antigen protein production. Recombinant Histidine (His)-tagged N-terminally truncated SARS-CoV-2-NP (121-419: ΔN-NP) was produced in a wheat germ cell-free system, and then purified using nickel-chelated Sepharose beads. Each protein fraction was analyzed by SDS-PAGE and visualized by CBB staining. Red arrow indicate the target protein. NC, Negative Control; T, Total fraction; S, Supernatant; P, Precipitate; FT, Flow-Through; E1–3, Elution fractions 1-3. (B) Flag-GST-tagged NPs derived from several human coronaviruses were produced in the wheat germ cell-free system. Reactivity of generated mAbs was validated by immunoblot analysis using the indicated antibodies.

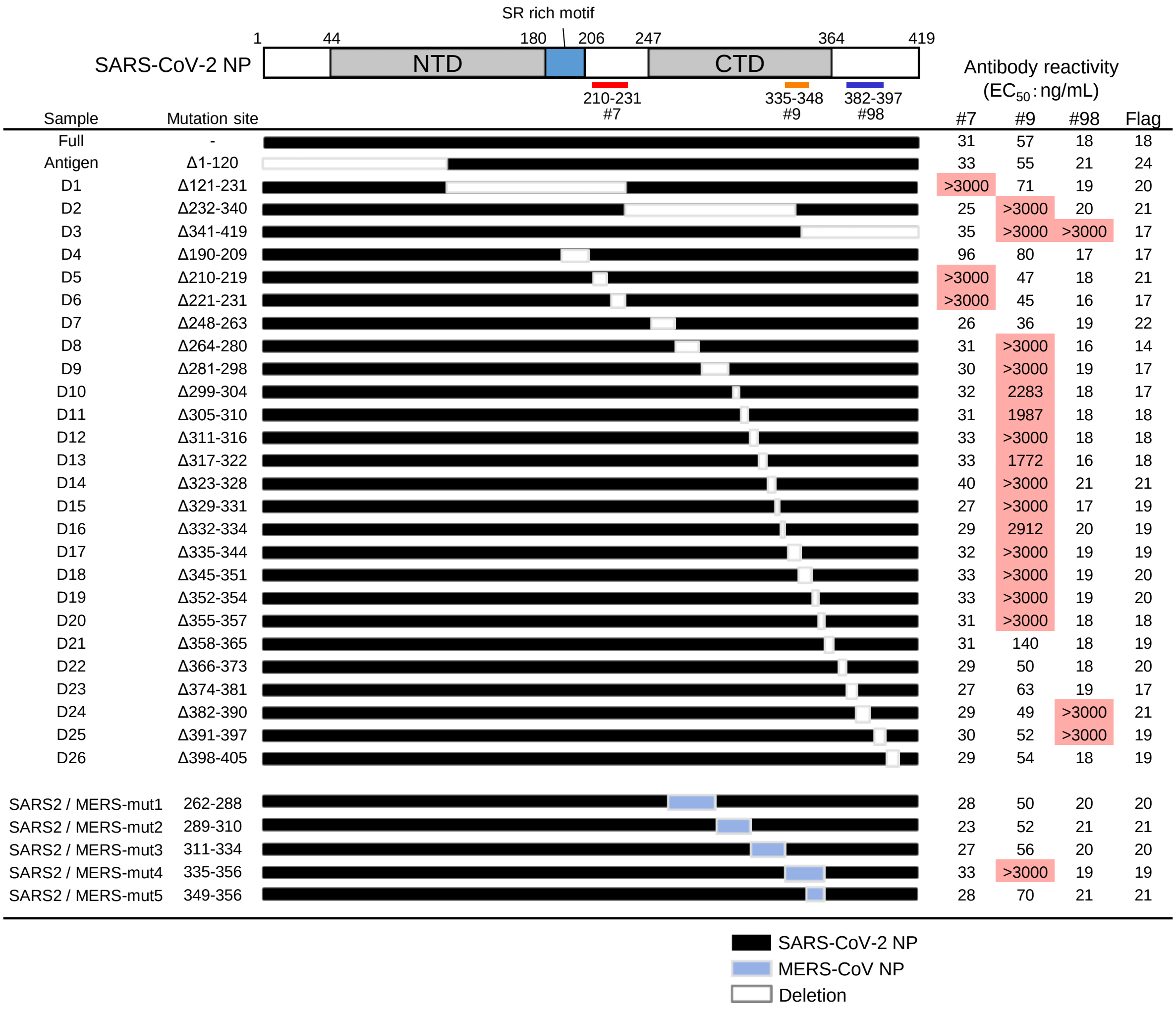

Sample	Mutation site	#7	#9	#98	Flag
Full	-	31	57	18	18
Antigen	Δ1-120	33	55	21	24
D1	Δ121-231	>3000	71	19	20
D2	Δ232-340	25	>3000	20	21
D3	Δ341-419	35	>3000	>3000	17
D4	Δ190-209	96	80	17	17
D5	Δ210-219	>3000	47	18	21
D6	Δ221-231	>3000	45	16	17
D7	Δ248-263	26	36	19	22
D8	Δ264-280	31	>3000	16	14
D9	Δ281-298	30	>3000	19	17
D10	Δ299-304	32	2283	18	17
D11	Δ305-310	31	1987	18	18
D12	Δ311-316	33	>3000	18	18
D13	Δ317-322	33	1772	16	18
D14	Δ323-328	40	>3000	21	21
D15	Δ329-331	27	>3000	17	19
D16	Δ332-334	29	2912	20	19
D17	Δ335-344	32	>3000	19	19
D18	Δ345-351	33	>3000	19	20
D19	Δ352-354	33	>3000	19	20
D20	Δ355-357	31	>3000	18	18
D21	Δ358-365	31	140	18	19
D22	Δ366-373	29	50	18	20
D23	Δ374-381	27	63	19	17
D24	Δ382-390	29	49	>3000	21
D25	Δ391-397	30	52	>3000	19
D26	Δ398-405	29	54	18	19
SARS2 / MERS-mut1	262-288	28	50	20	20
SARS2 / MERS-mut2	289-310	23	52	21	21
SARS2 / MERS-mut3	311-334	27	56	20	20
SARS2 / MERS-mut4	335-356	33	>3000	19	19
SARS2 / MERS-mut5	349-356	28	70	21	21

Supplementary Figure 2. (Related to Figure 1) Epitope mapping of mAbs.

Schematic diagram of SARS-CoV-2-NP domain architecture, mutants, and region of determined epitope of each monoclonal antibody. For epitope mapping, 26 deletion mutants (D1-D26) and 5 substitution mutants (SARS2/MERS-mut1-5) were produced as FLAG-GST-tagged proteins in the wheat germ cell-free system. NTD, N-terminal domain; LKR, flexible linker region; CTD, C-terminal domain. Reactivity of each mAb to deletion mutants was evaluated by indirect-ELISA. EC_{50} was calculated using six different antibody concentrations, and samples with an over 5-fold increase in EC_{50} compared to full-length NP were defined as negative (Red).

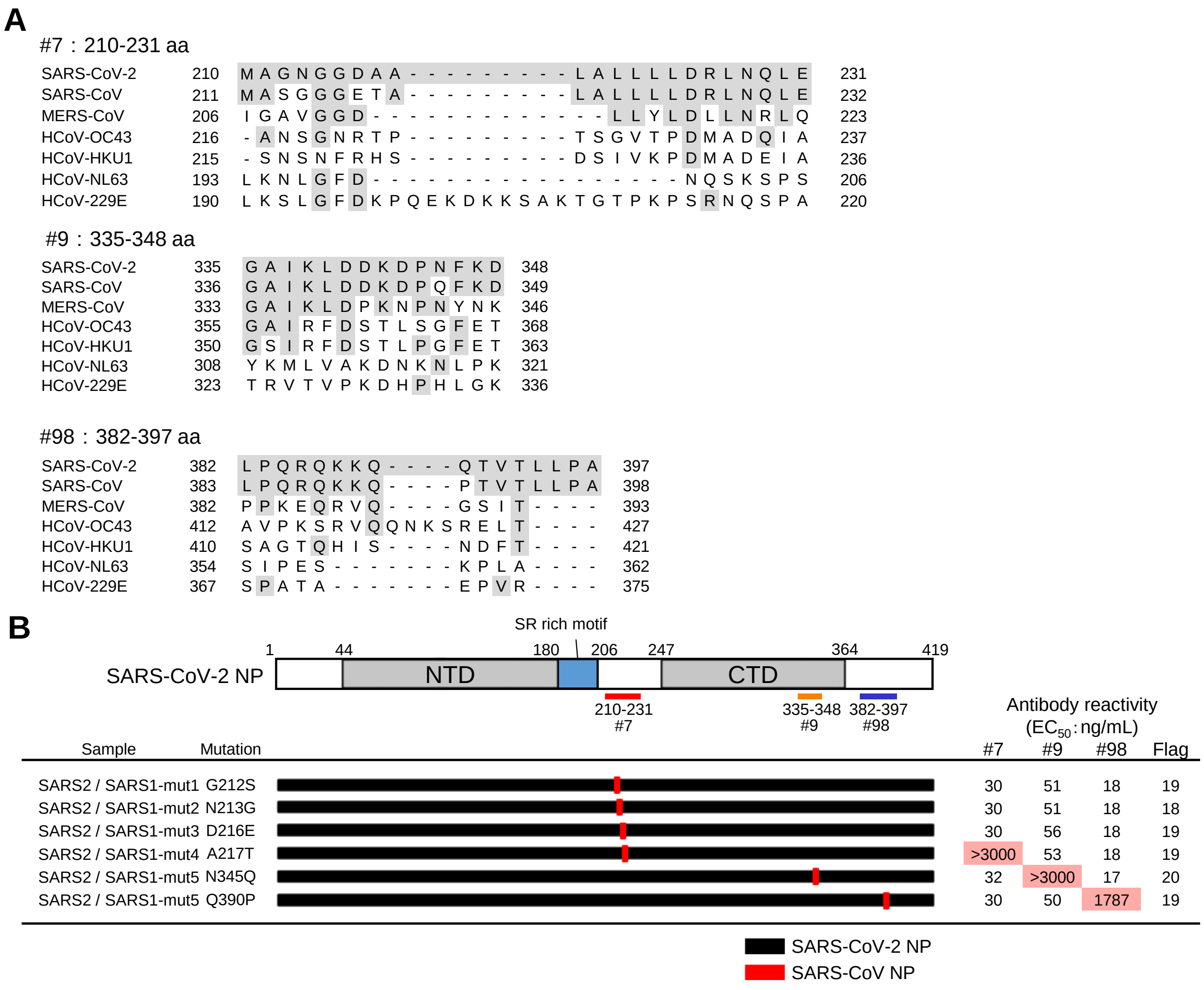

Supplementary Figure 3. (Related to Figure 1) Critical residues for desecting SARS-CoV and SARS-CoV-2 in epitope of developed mAbs.

(A) Multiple sequence alignments of NP of human coronaviruses in the epitope region using MUSCLE software. Gray shaded positions represent conserved residues among the sequences. (B) Determination of critical residues for specific detection of not SARS-CoV but SARS-CoV-2. Five point substitution mutants (SARS2/SARS1-mut1-5) were produced as FLAG-GST-tagged proteins in the wheat germ cell-free system. NTD, N-terminal domain; LKR, flexible linker region; CTD, C-terminal domain. Reactivity of each mAb to deletion mutants was evaluated by indirect-ELISA. EC_{50} was calculated using six different antibody concentrations. The samples with an over 5-fold increase in EC_{50} compared to full-length NP were defined as negative (Red).

A

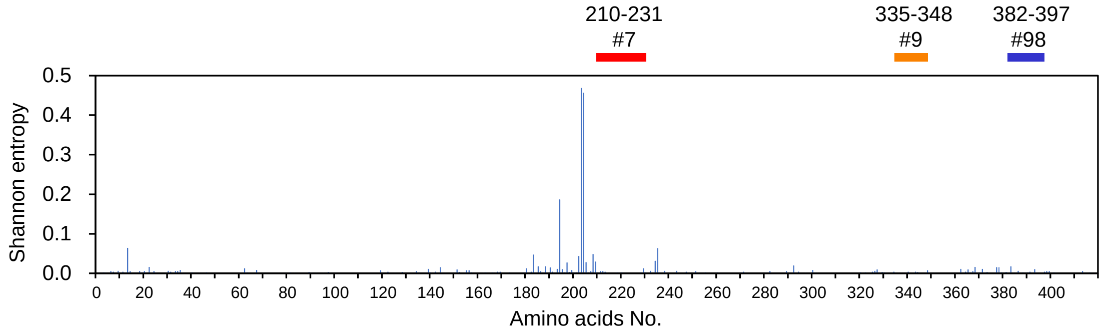

B

#7：210-231 aa

PANGO lineage of SARS-CoV-2		Amino acid sequence of epitope	
A (Prototype)	210	M A G N G G D A A L A L L L L D R L N Q L E	231
B.1.1.7 (501Y.V1)	210	M A G N G G D A A L A L L L L D R L N Q L E	231
B.1.351 (501Y.V2)	210	M A G N G G D A A L A L L L L D R L N Q L E	231
B.1.427	210	M A G N G G D A A L A L L L L D R L N Q L E	231
B.1.429	210	M A G N G G D A A L A L L L L D R L N Q L E	231
B.1.525	210	M A G N G G D A A L A L L L L D R L N Q L E	231
B.1.526	210	M A G N G G D A A L A L L L L D R L N Q L E	231
P.1 (501Y.V3)	210	M A G N G G D A A L A L L L L D R L N Q L E	231
P.2	210	M A G N G G D A A L A L L L L D R L N Q L E	231

#9：335-348 aa

PANGO lineage of SARS-CoV-2		Amino acid sequence of epitope	
A (Prototype)	335	G A I K L D D K D P N F K D	348
B.1.1.7 (501Y.V1)	335	G A I K L D D K D P N F K D	348
B.1.351 (501Y.V2)	335	G A I K L D D K D P N F K D	348
B.1.427	335	G A I K L D D K D P N F K D	348
B.1.429	335	G A I K L D D K D P N F K D	348
B.1.525	335	G A I K L D D K D P N F K D	348
B.1.526	335	G A I K L D D K D P N F K D	348
P.1 (501Y.V3)	335	G A I K L D D K D P N F K D	348
P.2	335	G A I K L D D K D P N F K D	348

#98：382-397 aa

PANGO lineage of SARS-CoV-2		Amino acid sequence of epitope	
A (Prototype)	382	L P Q R Q K K Q Q T V T L L P A	397
B.1.1.7 (501Y.V1)	382	L P Q R Q K K Q Q T V T L L P A	397
B.1.351 (501Y.V2)	382	L P Q R Q K K Q Q T V T L L P A	397
B.1.427	382	L P Q R Q K K Q Q T V T L L P A	397
B.1.429	382	L P Q R Q K K Q Q T V T L L P A	397
B.1.525	382	L P Q R Q K K Q Q T V T L L P A	397
B.1.526	382	L P Q R Q K K Q Q T V T L L P A	397
P.1 (501Y.V3)	382	L P Q R Q K K Q Q T V T L L P A	397
P.2	382	L P Q R Q K K Q Q T V T L L P A	397

Supplementary Figure 4. (Related to Figure 1) Shannon entropy analysis of nucleocapsid proteins and comparison of epitope sequences between prototype and current variant of SARS-CoV-2.

(A) Shannon entropy, as a quantitative measure of variation, was calculated for each amino-acid residue of SARS-CoV-2 NP derived from 8,127 strains. Binding sites of each mAb are indicated in different colors. **(B)** Amino acid sequence in the antigenic epitope of reference strain against each of the currently reported epidemic variant of SARS-CoV-2 [11]. Gray shaded positions represent conserved residues among the sequences.

A

#7：210-231 aa

ACCESSION ID	Name of strain	Number of strains	Relative abundance	210	211	212	213	214	215	216	217	218	219	220	221	222	223	224	225	226	227	228	229	230	231
NC_045512	Reference genome	8091	99.56%	M	A	G	N	G	G	D	A	A	L	A	L	L	L	L	D	R	L	N	Q	L	E
MT847220	BGD/BCSIR NILMRC 281/2020	3	0.04%	.	S	.	.	.	.	.	.	.	.	.	.	.	.	.	.	.	.	.	.	.	.
MT706376	USA/WI-UW-249/2020	1	0.01%	I	.	.	.	.	.	.	.	.	.	.	.	.	.	.	.	.	.	.	.	.	.
MT831837	USA/WA-S2367/2020	1	0.01%	V	.	.	.	.	.	.	.	.	.	.	.	.	.	.	.	.	.	.	.	.	.
MT358640	DEU/FFM4/2020	2	0.02%	.	.	.	.	.	.	.	.	.	.	.	.	.	.	.	.	.	.	.	.	F	.
MT873396	USA/MA-MGH-01121/2020	1	0.01%	.	V	.	.	.	.	.	.	.	.	.	.	.	.	.	.	.	.	.	.	.	.
MT628169	USA/CA-CZB-1484/2020	2	0.02%	.	.	.	.	.	.	.	.	V	.	.	.	.	.	.	.	.	.	.	.	.	.
MT472626	USA/IA-CDC-8200/2020	2	0.02%	.	.	.	.	.	.	.	.	.	.	T	.	.	.	.	.	.	.	.	.	.	.
MT601287	BGD/BCSIR NILMRC 75/2020	13	0.16%	.	.	.	.	.	.	.	.	.	.	.	.	.	.	.	.	.	.	.	H	.	.
MT535490	USA/UT-01568/2020	2	0.02%	.	.	V	.	.	.	.	.	.	.	.	.	.	.	.	.	.	.	.	.	.	.
MT614522	USA/WA-QDX-62/2020	2	0.02%	.	.	C	.	.	.	.	.	.	.	.	.	.	.	.	.	.	.	.	.	.	.
MT252734	USA/WA-UW154/2020	4	0.05%	.	.	.	Y	.	.	.	.	.	.	.	.	.	.	.	.	.	.	.	.	.	.
MT451476	AUS/VIC680/2020	2	0.02%	.	.	.	.	.	.	.	.	.	.	.	.	.	.	.	.	.	.	Y	.	.	.
MT806787	USA/IN-QDX-251/2020	1	0.01%	.	.	.	.	C	.	.	.	.	.	.	.	.	.	.	.	.	.	.	.	.	.

#9：335-348 aa

ACCESSION ID	Name of strain	Number of strains	Relative abundance	335	336	337	338	339	340	341	342	343	344	345	346	347	348
NC_045512	Reference genome	8107	99.75%	G	A	I	K	L	D	D	K	D	P	N	F	K	D
MT873324	USA/MA-MGH-01405/2020	1	0.01%	.	S	.	.	.	.	.	.	.	.	.	.	.	.
MT706363	USA/WI-UW-235/2020	1	0.01%	.	.	F	.	.	.	.	.	.	.	.	.	.	.
MT326140	USA/WA-UW-1680/2020	2	0.02%	.	.	.	.	.	N	.	.	.	.	.	.	.	.
MT831745	USA/WA-S2426/2020	1	0.01%	.	.	.	.	.	.	.	.	.	.	T	.	.	.
LR877181	SWE/STO-KI/2020	1	0.01%	.	.	.	N	.	.	.	.	.	.	.	.	.	.
MT683418	USA/WA-UW-10138/2020	1	0.01%	.	.	.	.	.	.	.	N	.	.	.	.	.	.
LC529905	JP/TKYE6182/2020	3	0.04%	.	.	.	.	.	.	.	.	.	S	.	.	.	.
MT358737	USA/WA-UW-3994/2020	1	0.01%	.	.	.	.	.	G	.	.	.	.	.	.	.	.
MT509498	IND/GBRC107/2020	4	0.05%	.	.	.	.	.	.	.	.	.	.	.	.	.	Y
MT731292	MAR/RMPS02/2020	2	0.02%	.	.	.	.	.	.	.	.	.	.	.	.	.	H
MT135043	CHN/233/2020	1	0.01%	.	.	.	.	.	.	.	.	V	.	.	.	.	.
MT811401	USA/SEARCH-0834-IPL/2020	2	0.02%	.	.	.	.	.	.	.	.	Y	.	.	.	.	.

#98：382-397 aa

ACCESSION ID	Name of strain	Number of strains	Relative abundance	382	383	384	385	386	387	388	389	390	391	392	393	394	395	396	397
NC_045512	Reference genome	8079	99.41%	L	P	Q	R	Q	K	K	Q	Q	T	V	T	L	L	P	A
MT811458	USA/SEARCH-0997-IPL/2020	1	0.01%	.	.	.	.	.	.	R	.	.	.	.	.	.	.	.	.
MT252716	USA/WA-UW180/2020	2	0.02%	.	.	.	.	.	.	.	.	.	.	.	.	.	.	.	S
MT614463	USA/CA-QDX-40/2020	1	0.01%	.	.	.	.	K	.	.	.	.	.	.	.	.	.	.	.
LC571012	JP/DP0654/2020	1	0.01%	.	.	.	.	.	.	.	.	.	.	.	.	.	.	.	V
MT831158	USA/WA-S2249/2020	5	0.06%	.	.	.	.	H	.	.	.	.	.	.	.	.	.	.	.
MT252737	USA/WA-UW146/2020	2	0.02%	.	.	.	.	.	.	.	.	.	I	.	.	.	.	.	.
MT358637	IND/GBRC1/2020	11	0.14%	.	.	.	.	.	.	.	.	.	.	.	I	.	.	.	.
MT757020	USA/FL-BPHL-0351/2020	1	0.01%	.	.	H	.	.	.	.	.	.	.	.	.	.	.	.	.
MT451616	AUS/VIC879/2020	9	0.11%	.	S	.	.	.	.	.	.	.	.	.	.	.	.	.	.
MT646100	USA/MD-HP00086/2020	9	0.11%	.	L	.	.	.	.	.	.	.	.	.	.	.	.	.	.
MT831179	USA/WA-S2105/2020	1	0.01%	S	.	.	.	.	.	.	.	.	.	.	.	.	.	.	.
MT825091	IRN/COVID19-IRVSH1/2020	1	0.01%	.	.	.	.	.	.	.	.	L	.	.	.	.	.	.	.
MT811500	USA/SEARCH-1065-SAN/2020	1	0.01%	.	.	.	.	.	.	I	.	.	.	.	.	.	.	.	.
MT757121	USA/FL-BPHL-0313/2020	1	0.01%	.	.	.	I	.	.	.	.	.	.	.	.	.	.	.	.
MT810952	USA/SEARCH-0240-SAN/2020	1	0.01%	.	.	.	.	.	.	.	.	.	.	.	.	.	.	H	.
MT434815	USA/NY-CDC-SURV0175NYC/2020	1	0.01%	.	.	.	.	.	.	.	.	-	-	-	.	.	.	.	.

B

#7：210-231 aa

Name	Mutation site	Antibody reactivity (EC_{50}: ng/mL) #7	#9	#98	Flag
Full	-	31	57	18	18
M1	A211S	28	51	17	20
M2	M210I	31	54	18	20
M3	M210V	30	55	18	20
M4	L230F	25	54	19	20
M5	A211V	31	57	18	20
M6	A218V	40	79	19	20
M7	A220T	29	58	18	20
M8	Q229H	27	56	19	21
M9	G212V	36	64	18	16
M10	G212C	153	77	18	17
M11	N213Y	51	74	18	17
M12	N228Y	56	89	17	18
M13	G214C	185	70	17	18

#9：335-348 aa

Name	Mutation site	Antibody reactivity (EC_{50}: ng/mL) #7	#9	#98	Flag
Full	-	31	57	18	18
M13	A336S	29	57	18	18
M14	I337F	29	62	18	19
M15	D340N	30	56	18	18
M16	N345T	30	63	17	19
M17	K338N	30	57	18	20
M18	K342N	30	52	18	18
M19	P344S	31	2658	16	19
M20	D340G	32	77	17	19
M21	D348Y	32	2348	16	18
M22	D348H	31	1755	17	18
M23	D343V	30	>3000	17	18
M24	D343Y	31	2316	16	17

#98：382-397 aa

Name	Mutation site	Antibody reactivity (EC_{50}: ng/mL) #7	#9	#98	Flag
Full	-	31	57	18	18
M25	K388R	30	52	16	18
M26	A397S	28	53	16	18
M27	Q386K	29	56	16	18
M28	A397V	29	54	15	19
M29	Q386H	28	54	17	18
M30	T391I	30	52	16	19
M31	T393I	28	50	>3000	19
M32	Q384H	29	56	18	19
M33	P383S	31	63	17	20
M34	P383L	31	58	18	21
M35	L382S	30	60	18	21
M36	Q390L	31	60	98	20
M37	K388I	30	60	19	21
M38	R385I	31	58	18	22
M39	P396H	30	58	15	20
M40	Δ390-392	29	53	>3000	21

Supplementary Figure 5. (Related to Figure 1) Reactivity of developed monoclonal antibodies against recombinant protein from divergent strains.

(A) Identification of amino-acid substitution in the antigenic epitope of each identical strain from 8,127 clinical strains compared with the reference strain. Dots indicate sequence identity relative to the reference strain. Gray shaded

positions represent conserved residues among the sequences. (B) Reactivity of mAbs to recombinant NP from strains possessing amino-acid substitution in the epitope regions. Forty point substitution mutants identified in (A) were produced as FLAG-GST-tagged proteins in the wheat germ cell-free system. Reactivity of each mAb to mutants was evaluated by indirect-ELISA. EC_{50} was calculated using six different antibody concentrations. The samples with an over 5-fold increase in EC_{50} compared to full-length NP were defined as negative (Red).

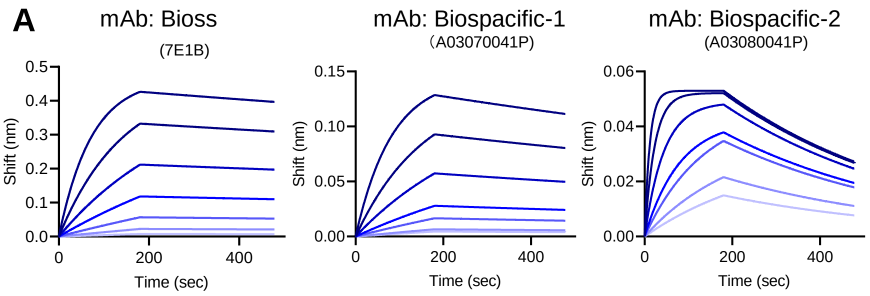

	K_D (M)	k_{on} (1/Ms)	k_{dis} (1/s)
Bioss (7E1B)	9.0E-10	2.7E+05	2.4E-04
Biospacific-1 (A03070041P)	1.2E-09	1.7E+05	2.1E-04
Biospacific-2 (A03080041P)	2.0E-09	1.9E+06	3.6E-03

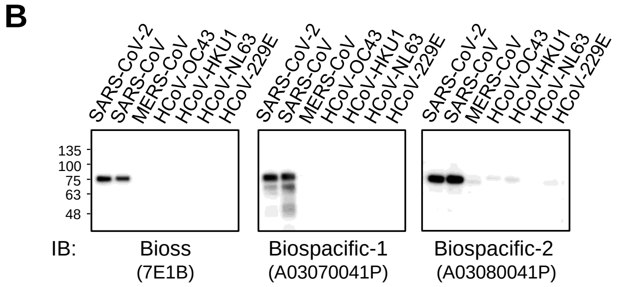

Supplementary Figure 6. (Related to Figure 2) Comparison of commercially available existing mAbs against SARS-CoV-2 NP.

(A) Affinity measurement of commercially available monoclonal antibodies on the octetRED96 instrument. Association and dissociation of each mAbs to full-length NP at various concentrations (50, 25, 12.5, 6.25, 3.13, 1.56 and 0.78 nM) was evaluated using AMC sensor. (B) Specificity of existing mAbs were examined by immunoblot analysis using the indicated recombinant proteins from human coronaviruses.

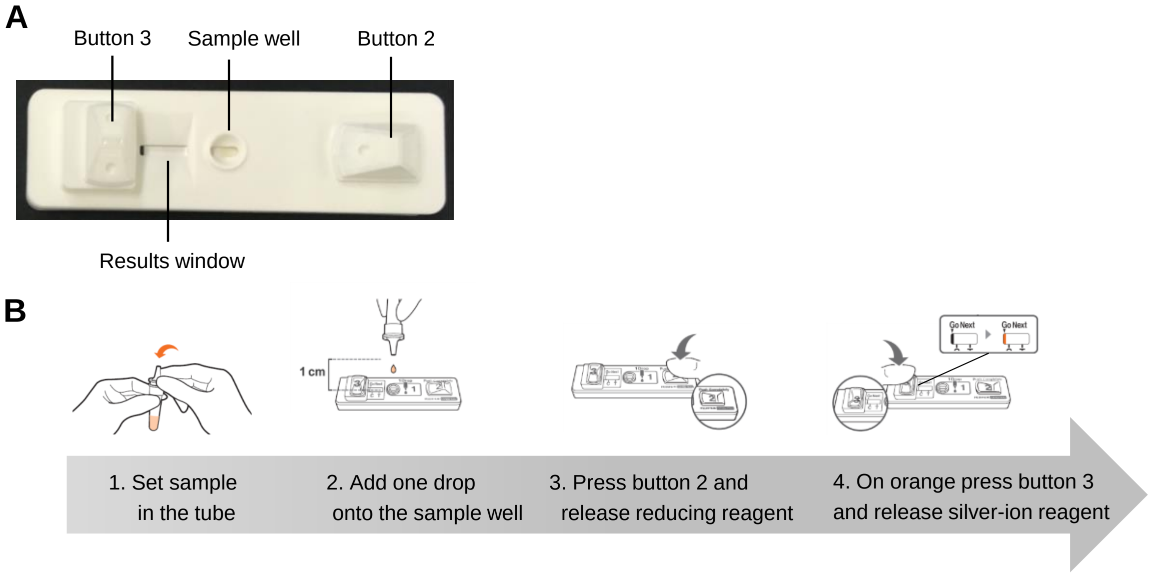

Supplementary Figure 7. (Related to Figure 4) Overview and procedure of LFIA.

(A) Image of LFIA Cartridge. (B) Samples were diluted with an extraction buffer (Tris buffer containing 0.1% non-ionic surfactant) in the tube and one drop of the sample was added onto the sample well of LFIA cartridge. Following this, button 2 was immediately pressed to release a reducing reagent for silver amplification. After the color indicator mark turned orange (about 10 min), button 3 was pressed to release a silver-ion reagent to activate the silver amplification reaction.

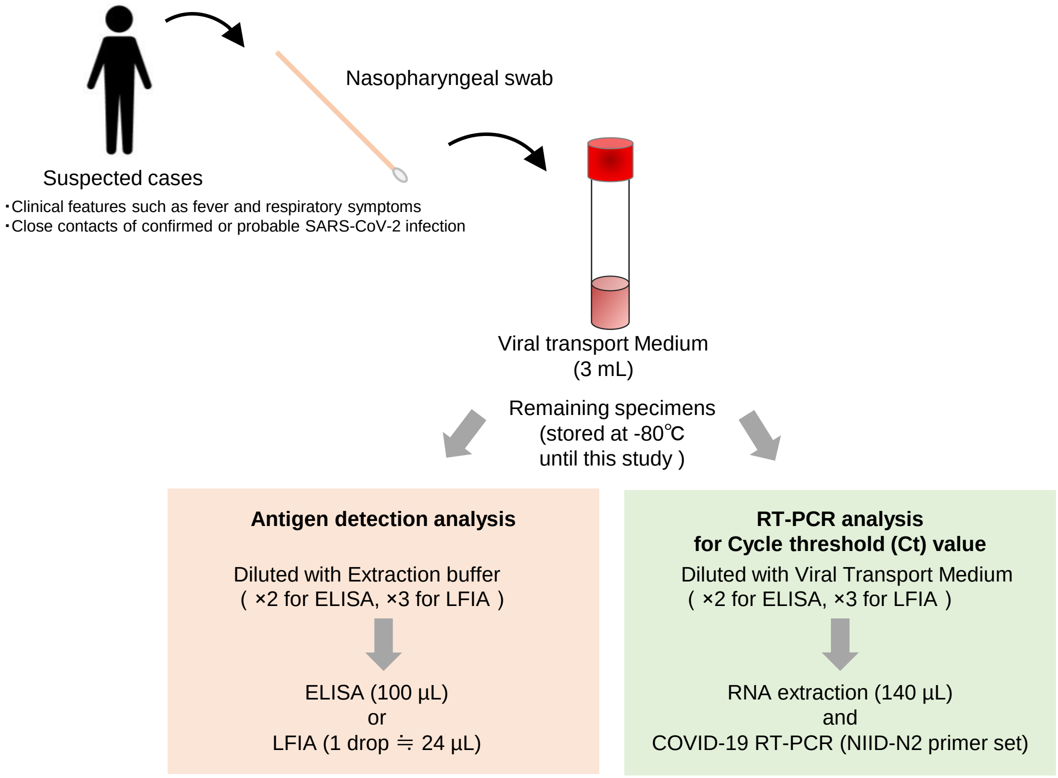

Supplementary Figure 8. (Related to STAR Methods) Evaluation of clinical specimens for antigen detection assay or RT-PCR.

Clinical specimens of nasopharyngeal swabs from cases with suspected SARS-CoV-2 infection were retrieved for this study. Swabs were collected in 3 mL of viral transport medium. RNA extraction and RT-PCR using NIID-N2 set primer were carried out as an administrative inspection. The remaining clinical samples were centrifuged, and the supernatant was used for this study. For antigen detection assays, samples were diluted 2-fold (ELISA) or 3-fold (LFIA) in extraction buffer and 100 µL (ELISA) or 1 drop (LFIA) was subjected to analysis. For RT-PCR analysis to quantify Ct value, samples were diluted to same concentration used in antigen detection assay (2-fold for ELISA, 3-fold for LFIA) with viral transport medium. RNA extraction and RT-PCR analysis were carried out using 140 µL of diluted samples.
