# Supplementary material for: Highly specific monoclonal antibodies and epitope identification against SARS-CoV-2 nucleocapsid protein for antigen detection tests
Source: Cell Rep Med. 2021 May 16;2(6):100311. doi: 10.1016/j.xcrm.2021.100311 (PMC8126173; doi:10.1016/j.xcrm.2021.100311)
Supplement: Document S2. Article plus supplemental information [file mmc2.pdf]

Cell Reports
Medicine

Article

Highly specific monoclonal antibodies and epitope identification against SARS-CoV-2 nucleocapsid protein for antigen detection tests

Graphical abstract

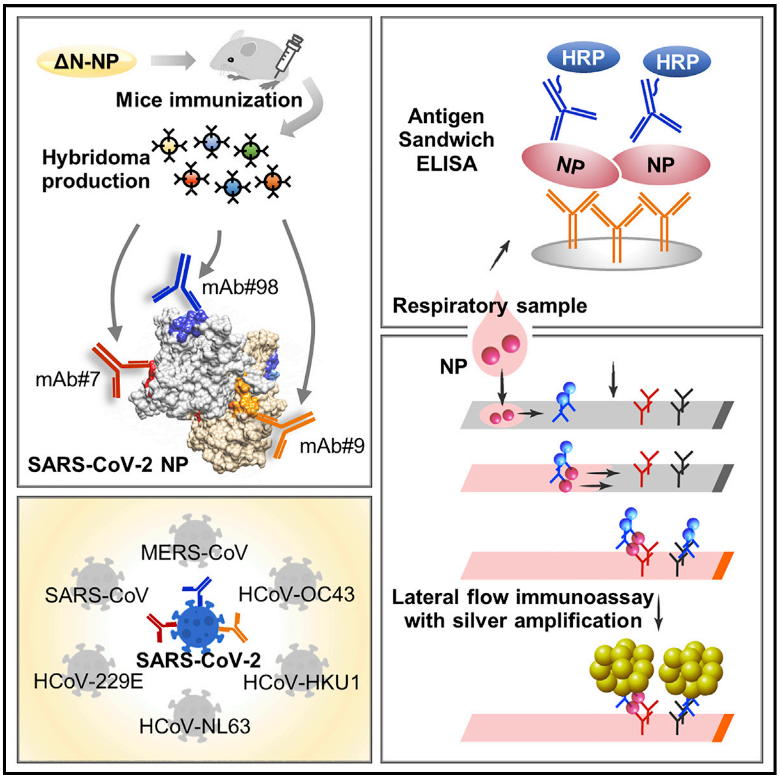

Highlights

- The monoclonal antibodies of this study have distinct epitopes to detect SARS-CoV-2
- They exhibit no cross-reactivity with other viruses, including SARS-CoV
- These antibodies accurately detect the major SARS-CoV-2 variants of concern
- Coupling rapid antigen test with silver amplification enhances the test performance

Authors

Yutaro Yamaoka, Kei Miyakawa, Sundararaj Stanleyraj Jeremiah, ..., Nobuhiko Okabe, Hideki Hasegawa, Akihide Ryo

Correspondence

aryo@yokohama-cu.ac.jp

In brief

In this study, Yamaoka et al. report their highly specific, epitope-characterized monoclonal antibodies that exclusively detect SARS-CoV-2. These monoclonal antibodies, when used in a lateral flow immunoassay coupled with silver amplification, enhance the performance of rapid antigen detection tests for COVID-19.

Yamaoka et al., 2021, Cell Reports Medicine 2, 100311
June 15, 2021 © 2021 The Authors.
https://doi.org/10.1016/j.xcrm.2021.100311

CellPress

Cell Reports Medicine

CellPress
OPEN ACCESS

Article

Highly specific monoclonal antibodies and epitope identification against SARS-CoV-2 nucleocapsid protein for antigen detection tests

Yutaro Yamaoka,[1,2,14] Kei Miyakawa,[1,14] Sundararaj Stanleyraj Jeremiah,[1] Rikako Funabashi,[1] Koji Okudela,[3] Sayaka Kikuchi,[2] Junichi Katada,[4] Atsuhiko Wada,[4] Toshiki Takei,[4] Mayuko Nishi,[1] Kohei Shimizu,[5] Hiroki Ozawa,[5] Shuzo Usuku,[5] Chiharu Kawakami,[5] Nobuko Tanaka,[5] Takeshi Morita,[1] Hiroyuki Hayashi,[6] Hideaki Mitsui,[3] Keita Suzuki,[2] Daisuke Aizawa,[2] Yukihiro Yoshimura,[7] Tomoyuki Miyazaki,[8] Etsuko Yamazaki,[9] Tadaki Suzuki,[10] Hirokazu Kimura,[11] Hideaki Shimizu,[12] Nobuhiko Okabe,[12] Hideki Hasegawa,[13] and Akihide Ryo[1,15,*]

[1]Department of Microbiology, Yokohama City University School of Medicine, Yokohama, Kanagawa 236-0004, Japan
[2]Life Science Laboratory, Technology and Development Division, Kanto Chemical Co., Inc., Isehara, Kanagawa 259-1146, Japan
[3]Department of Pathology, Yokohama City University Graduate School of Medicine, Yokohama, Kanagawa 236-0004, Japan
[4]Medical Systems Research & Development Center, FUJIFILM Corporation, Kaisei, Kanagawa 258-8538, Japan
[5]Yokohama City Institute of Public Health, Yokohama, Kanagawa 236-0051, Japan
[6]Division of Pathology, Yokohama Municipal Citizen's Hospital, Yokohama, Kanagawa 221-0855, Japan
[7]Division of Infectious Disease, Yokohama Municipal Citizen's Hospital, Yokohama, Kanagawa 221-0855, Japan
[8]Department of Physiology, Yokohama City University Graduate School of Medicine, Yokohama, Kanagawa 236-0004, Japan
[9]Clinical Laboratory Department, Yokohama City University Hospital, Yokohama, Kanagawa 236-0004, Japan
[10]Department of Pathology, National Institute of Infectious Diseases, Shinjuku, Tokyo 162-8640, Japan
[11]School of Medical Technology, Faculty of Health Sciences, Gunma Paz University, Takasaki, Gunma 370-0006, Japan
[12]Division of Virology, Kawasaki City Institute for Public Health, Kawasaki, Kanagawa 210-0821, Japan
[13]Center for Influenza and Respiratory Virus Research, National Institute of Infectious Diseases, Musashimurayama, Tokyo 208-0011, Japan
[14]These authors contributed equally
[15]Lead contact
*Correspondence: aryo@yokohama-cu.ac.jp
https://doi.org/10.1016/j.xcrm.2021.100311

SUMMARY

The ongoing coronavirus disease 2019 (COVID-19) pandemic is a major global public health concern. Although rapid point-of-care testing for detecting viral antigen is important for management of the outbreak, the current antigen tests are less sensitive than nucleic acid testing. In our current study, we produce monoclonal antibodies (mAbs) that exclusively react with severe acute respiratory syndrome coronavirus 2 (SARS-CoV-2) and exhibit no cross-reactivity with other human coronaviruses, including SARS-CoV. Molecular modeling suggests that the mAbs bind to epitopes present on the exterior surface of the nucleocapsid, making them suitable for detecting SARS-CoV-2 in clinical samples. We further select the optimal pair of anti-SARS-CoV-2 nucleocapsid protein (NP) mAbs using ELISA and then use this mAb pair to develop immunochromatographic assay augmented with silver amplification technology. Our mAbs recognize the variants of concern (501Y.V1-V3) that are currently in circulation. Because of their high performance, the mAbs of this study can serve as good candidates for developing antigen detection kits for COVID-19.

INTRODUCTION

Coronavirus disease 2019 (COVID-19), the disease caused by severe acute respiratory syndrome coronavirus 2 (SARS-CoV-2), happens to be the most recent threat to mankind causing major public health issues across the world.[1] Breaking out of all containment efforts, the virus has spread across international borders to cause a massive pandemic.[2] Urgent measures are required to tackle this outbreak, as countries worldwide have reported over 127 million infections and 2.7 million deaths.[3] As the complete details about the nature and pathogenicity of the virus still remain enigmatic, it would be only wise to limit new infections to as low as possible.

Despite the urgency of the situation, rapid diagnostic methods and point-of-care testing (POCT) that can be used to make immediate and on-site diagnostic decisions have not been convincingly established.[4] Current diagnostic methods for COVID-19 are deployed after medical examination for the presence of clinical features, such as fever and cough and history of exposure or travel. This strategy fails to detect the asymptomatically infected people who could act as an unidentified source to propagate the disease in the community.[5] At present, the recommended methods to establish a diagnosis of COVID-19 principally employ nucleic acid amplification tests (NATs), such as reverse-transcriptase polymerase chain reaction (RT-PCR) or reverse-transcription loop-mediated isothermal amplification

This is an open access article under the CC BY license (http://creativecommons.org/licenses/by/4.0/).

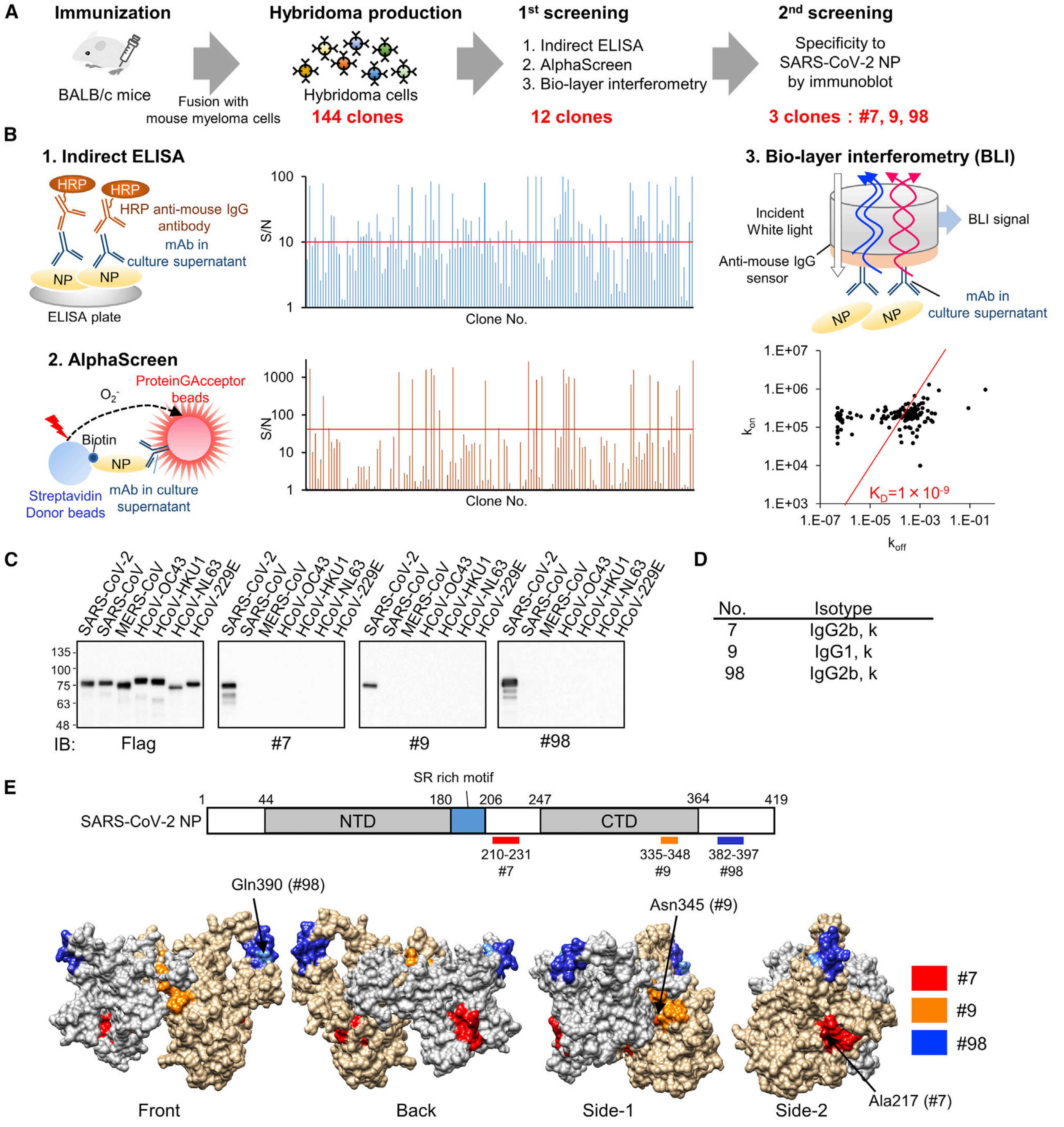

No.	Isotype
7	IgG2b, k
9	IgG1, k
98	IgG2b, k

Figure 1. Production of high-affinity and specific mAb against SARS-CoV-2 NP using wheat germ cell-free synthesized antigen

(A) Schematic diagram of hybridoma cells production to generate anti-SARS-CoV-2-NP mAb. Purified protein was injected into BALB/c mice. After 4 weeks, lymphocytes from immunized mice were fused with myeloma cells, and 144 hybridoma cells were established.

(B) Of the 144 clones, the 12 that exhibited high reactivity to antigen proteins, as revealed by indirect ELISA, AlphaScreen, and Bio-layer interferometry, were selected for further investigation (two technical replicates). Red line indicates cutoff line of screening; S/N = 10 for ELISA, S/N = 40 for AlphaScreen, and K_D = 1.0×10^9. k_{on} and k_{off} values for each antibody clones to the ΔN-NP antigen estimated by OctetRED96 instrument using hybridoma supernatant are indicated as dots on the two-dimensional plot.

(C) Specificity screening of mAbs. FLAG-glutathione S-transferase (GST)-tagged NPs derived from several human coronaviruses were produced in the wheat germ extract system. Reactivity of generated mAbs was validated by immunoblot analysis using either anti-FLAG or the indicated antibodies. Three clones specifically detect SARS-CoV-2 NP and were selected (representative data of two technical replicates).

(D) Isotype of selected mAbs.

(legend continued on next page)

(RT-LAMP), which are difficult to scale up for performing on a multitude of patients at the clinical sites.[6,7] This underscores the need for a de-centralized, simple, reliable, and rapid POCT for diagnosing COVID-19 at a mass scale.

Viral antigen detection is a convenient method to directly demonstrate SARS-CoV-2 in infected individuals as compared to NAT and can provide test results in a much shorter time span of under 30 min. The performance of an antigen-detection kit principally relies on employing high-quality monoclonal antibodies (mAbs) that precisely target specific viral antigens. Certain antigen-detection kits currently approved for the diagnosis of COVID-19 utilize mAbs against SARS-CoV to detect SARS-CoV-2.[8–10] This may result in the inadequate specificity as well as sensitivity because the epitope regions become less immunogenic due to amino acid substitutions between viral species. Furthermore, multiple viral variants with increasing infectivity and transmissibility have emerged continuously.[11] It has been reported that some genetic mutations may cause false-negative results in NAT.[12] Also, currently approved kits have not disclosed such information with detailed epitope analysis.[8,13]

Nucleocapsid protein (NP) is a viral antigen that organizes the single-stranded RNA into a helical capsid structure. NP is abundantly expressed during SARS-CoV-2 replication and is highly immunogenic, making it a suitable target for antigen testing.[14] This advantage, however, comes with a drawback of SARS-CoV-2 NP exhibiting high homology with the NP of other human coronaviruses, leading to the problem of cross-reactivity, which could be the reason for the unavailability of accurate antigen-detection tests. In addition, SARS-CoV-2 is almost identical to SARS-CoV (~90% identity), because of which none of the currently available mAbs discriminate SARS-CoV-2 NP from that of SARS-CoV.[15] Hence, it is a pressing demand to create mAbs that can exclusively target SARS-CoV-2 in order to establish a precise antigen-detection kit for reliable diagnosis of COVID-19.

The production of high-precision mAb is essentially determined by the quality of antigen before immunization.[16,17] Preparation of high-quality antigen is essential for generating specific mAbs that recognize the native form of the corresponding viral antigen.[16] The wheat germ cell-free protein production system is a sophisticated approach based on the eukaryotic translation apparatus of wheat seeds, with significant advantages over other commonly used protein expression systems for large-scale protein production.[18,19] Owing to the eukaryotic translation machinery, this system can synthesize properly folded and biologically active proteins identical to those expressed in mammalian cells.[20] These advantages highlight the suitability of this system for the generation of practically relevant antigenic proteins that can be used to immunize animals and generate mAbs. In our previous study, we were able to demonstrate favorable results upon producing NP of Middle-East severe respiratory syndrome (MERS) coronavirus using this system.[21]

In the current study, we have synthesized recombinant SARS-CoV-2 NP and produced mAbs that could specifically detect this protein. We also determined the optimal pair of the newly produced anti-NP mAbs to develop antigen capture ELISA and lateral flow immunochromatography assay (LFIA) for sensitive and definitive detection of SARS-CoV-2.

RESULTS

Production of mAbs specific for SARS-CoV-2 NP

NP has conserved amino acid (aa) residues at the N-terminal domain (NTD) (aas 1–120) that are homologous among human coronaviruses.[22] We produced N-terminal truncated nucleocapsid protein (ΔN-NP; aas 121–419) devoid of the homologous residues and used it as an immunogen to produce mAbs that are specific for SARS-CoV-2. His-tagged ΔN-NP was successfully expressed using wheat germ extracts and was purified as a soluble protein for immunization to BALB/c mice (Figure S1A). After 4 weeks of immunization, lymphocytes were harvested from the immunized mice and were fused with mouse myeloma cells to establish 144 stable hybridomas designated as nos. 1–144 (Figure 1A). The mAbs generated from these hybridomas were subjected to assess their reactivity to intact NP antigen by indirect ELISA, AlphaScreen, and Bio-Layer Interferometry (Figure 1B). Consequently, we selected 12 hybridoma clones that had high reactivity and affinity to the antigen in all three methods and next performed the immunoblot-based screening, where we sought to identify the mAbs that can specifically react with SARS-CoV-2 NP, but not with other human coronaviruses, including SARS-CoV. Of the 12 clones, three clones (no. 7, no. 9, and no. 98) were found to be completely specific to SARS-CoV-2 NP, exhibiting no cross-reactivity with other related viruses (Figure 1C), although the remaining clones showed cross-reactivity only to SARS-CoV (Figure S1B). Isotype analysis revealed that mAb no. 9 belonged to immunoglobulin G1 (IgG1) kappa isotype although both no. 7 and no. 98 belonged to IgG2b kappa isotype (Figure 1D).

Epitope analysis of mAbs

We next performed an ELISA-based epitope mapping to determine the antibody-binding sites using recombinant SARS-CoV-2 NP and its deletion mutants (Figure S2). We found that our newly developed mAbs recognized three distinct regions in NP: mAb no. 7 bound to 210–231 aas and mAb no. 98 bound to 382–397 aas (Figure 1E). Because mAb no. 9 did not react with many deletion mutants of C-terminal domain (CTD) (264–365 aas), we inferred that this problem could be due to the conformational changes due to inappropriate protein folding in deletion mutants. To overcome this trouble, we produced substitution mutants in which each region of the CTD was recombined with a homologous region of MERS-NP and examined the reactivity of the antibodies. With this design, ELISA analysis showed that mAb no. 9 bound to 335–348 aas (Figure S2).

(E) Schematic diagram of SARS-CoV-2-NP domain architecture and epitopes of antibodies. CTD, C-terminal domain; LKR, flexible linker region; NTD, N-terminal domain. Positions of epitopes in a structural model of whole-length dimer-forming NP, constructed by homology modeling using partial structures of SARS-CoV-2 NP are shown (PDB: 6yun; PDB: 6m3m). Epitope localizations of each mAb on molecular surface are highlighted in different colors. Critical residues for the specificity of mAb were indicated by arrows.

We next confirmed the antigenic discrimination ability of our newly developed mAbs. Multiple alignments of NPs derived from various human coronaviruses were carried out to examine the identity of epitope regions. Consistent with specificity analysis in Figure 1C, all the binding sites were found to be specific to SARS-CoV-2 (Figure S3A). The numbers of distinct aas contributing to specific recognition of mAb to SARS-CoV-2 NP, but not SARS-CoV NP, were four for mAb no. 7 and one each for mAbs no. 9 and no. 98. There were more than 5 aa differences in the antibody epitopes of other human coronaviruses (Figure S3A). To identify the critical residue of specificity in the epitope of each mAb, we created site-directed mutation with single-amino-acid substitution and examined the reactivity by ELISA. The results showed that Ala217 for no. 7, Asn345 for no. 9, and Gln390 for no. 98 were critical residues to discriminate SARS-CoV and SARS-CoV-2 (Figure S3B).

We next investigated whether the antigenic epitopes were located on the exterior surface of the nucleocapsid. Because the structure of full-length nucleocapsid protein has not been elucidated, homology modeling was carried out using previously reported partial structures of SARS-CoV-2 NP (PDB: 6M3M; PDB: 6YUN).[23,24] Molecular modeling of dimeric SARS-CoV-2 NP revealed that binding regions for all three mAbs were located on the surface of the protein (Figure 1E). Furthermore, these regions were spatially separated in the 3D structure, indicating the possibility to simultaneously use a pair of different mAbs to develop antigen-sandwich-detection assays.

Reactivity of antibodies to divergent strains of SARS-CoV-2

To estimate the reactivity of the mAbs with different clinical strains of SARS-CoV-2, we compared the aa sequences of NP derived from clinical isolates registered in the NCBI database. We collected 8,127 complete genomes that were registered in the NCBI database at the start of the study. Shannon entropy analysis revealed that the entire aa sequences of NP were highly conserved except for three high-frequency substitutions, S194L, R203K, and G204R, which were same as previously described (Figure S4A).[25] We observed that the epitope of each of the mAbs was completely conserved and devoid of any mutation in the current circulating mutants of SARS-CoV-2, including 501Y.V1, 501Y.V2, and 501Y.V3 (Figure S4B). Furthermore, all binding sites were highly conserved among the different strains of SARS-CoV-2 with more than 99% identity (Figure S5A). We next performed site-directed mutagenesis to introduce the binding site mutations into SARS-CoV-2 NP and examined the effect on the reactivity of mAbs by ELISA analysis (Figure S5B). Despite the mutations, our mAbs were able to bind to the mutated NPs at a rate of 99.9% for no. 7, 99.86% for no. 9, and 99.84% for no. 98.

Characterization of mAbs

The equilibrium dissociation constant (K_D) between selected antibodies and full-length NP of SARS-CoV-2 was determined by Bio-Layer interferometry. K_D for the antibody-antigen binding in mAbs nos. 7, 9, and 98 was calculated as 4.4×10^{-10}, 3.7×10^{-10}, and 1.6×10^{-10} respectively, all of which were lower than that of the commercially available mAbs (Figures 2A and S6A), and the latter showed cross-reactivity only to SARS-CoV NP (Figure S6B). These findings reveal that our mAbs had more specific and high-affinity binding to SARS-CoV-2 NP than the commercial antibodies.

We next performed immunoblot analysis with cell lysates from SARS-CoV-2-infected cells. All the three mAbs detected a 46-kDa protein band consistent with the molecular mass of SARS-CoV-2 NP (Figure 2B). We subsequently tested the applicability of these mAbs in immunofluorescence analysis using SARS-CoV-2-infected Vero E6-TMPRSS2 cells and found that no. 7 and no. 98 exhibited prominent and specific staining of the NP antigen in infected cells (Figure 2C). Additionally, mAb no. 98 was able to detect SARS-CoV-2 in infected cells in the paraffin-embedded lung tissue of a COVID-19 patient by immunohistochemical analysis (Figure 2D).

Development of antigen-capture ELISA

Because we had observed the different spatial binding of our mAbs in epitope localization analysis (Figure 1E), we exploited this feature to develop an antigen-capture sandwich ELISA (Figure 3A). Therefore, we evaluated the optimal pair of mAbs that can be employed for this purpose by testing all possible combinations of immobilized and labeled mAbs. The combination of no. 9 and no. 98 exhibited best signal-to-noise (S/N) ratios as compared to other pairs (Figure 3B). We used this combination to establish the SARS-CoV-2 NP antigen-capture ELISA with mAb no. 9 in the stationary phase and mAb no. 98 as the conjugate with horseradish peroxidase (HRP). We next determined the antigen detection threshold for this assay using serially diluted recombinant SARS-CoV-2 NP. The antigen-capture ELISA detected a volume of NP as low as 3.2 pg/mL (Figure 3C). SARS-CoV-2 virions inactivated by adding a detergent Nonidet P 40 (NP-40) were further examined by the ELISA system, and the detection limit of the assay was found to be 3.3×10^4 copies/mL (Figure 3C). Furthermore, we validated the specificity of our ELISA by testing simulated nasopharyngeal swab specimens prepared by adding either the recombinant NP of other human coronaviruses or virions of other human coronaviruses and common respiratory viruses (Figure 3D). Additionally, the ELISA was able to detect all the three major variants of concern (501Y.V1-3; Figure 3E).

We next evaluated the clinical performance of the ELISA assay using specimens that had been diagnosed by RT-PCR. For this purpose, we retrospectively selected 72 RT-PCR-negative and 72 RT-PCR-positive respiratory samples (nasopharyngeal swabs; Figure 3F). Using the respiratory samples, the cutoff values were determined with the area under receiver operating characteristic (ROC) curves where the specificity of the ELISA assay was set as 100.0% (95% confidence interval [CI]: 94.9%–100.0%) and the overall sensitivity was 75.0% (95% CI: 63.9%–83.6%; Figure 3G). Furthermore, positive respiratory samples were divided into three groups based on the Ct (threshold cycle) values as follows; samples with Ct values <28 were classified as “high viral load,” 28–31 were “intermediate viral load,” and samples >31 were “low viral load.” We observed that the clinical sensitivity of the ELISA varied depending on Ct value in RT-PCR and increased with the lower Ct. The clinical sensitivity at Ct < 28 was 100% (95% CI: 89.9%–100.0%), at Ct 28–31 was 92.3% (95% CI: 66.7%–99.6%), and at Ct > 31

CellPress
OPEN ACCESS

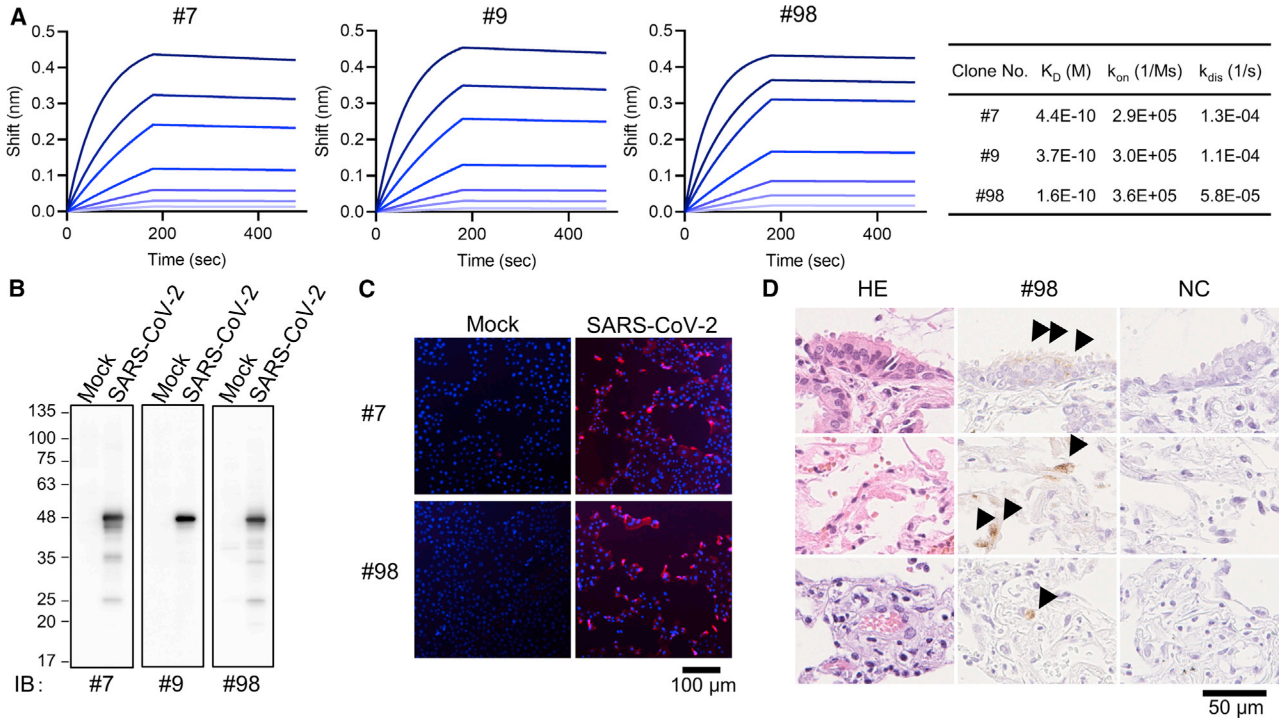

Figure 2. Binding affinity of developed mAbs and the application for viral NP antigen detection

(A) Affinity measurement of selected monoclonal antibodies on the Octet RED96 instrument. Association and dissociation of each mAb to full-length NP at various concentrations (50, 25, 12.5, 6.25, 3.13, 1.56, and 0.78 nM) was evaluated using anti-mouse IgG capture (AMC) sensor (two technical replicates).

(B) Immunoblot analysis of mock or SARS-CoV-2-infected VeroE6/TMPRSS2 cell lysates (representative image of two technical replicates).

(C) Immunofluorescence analysis (representative image of two technical replicates). VeroE6/TAMPRSS2 cells were infected or mock infected with SARS-CoV-2. After 24 h, cells were fixed and then stained with mAbs (hybridoma supernatant; red) and DAPI (blue).

(D) Paraffin-embedded lung biopsy specimen from a case of COVID-19 was examined for immunohistochemical detection of SARS-CoV2 using our antibody. The positive signals (arrowheads) are seen in bronchiolar epithelial cells (top), pneumocytes (middle), and endothelial cell (bottom).

was 32.0% (95% CI: 17.2%–51.6%; Figures 3H and 3I). The reactivity of antigen-capture ELISA correlated with RNA copy numbers in the specimens calculated based on the Ct values (Figure 3J).

Development of rapid lateral flow immunochromatographic assay

Finally, we examined whether the antibody set could be applied to LFIA, which could serve as a POCT device. We utilized silver halide photography technology for output signal amplification in our LFIA (Figures 4A, S7A, and S7B).[26,27] In this technology, silver ions are allowed to adhere to the surface of gold nanoparticles (approx. 0.05 μm in diameter), which allows the electrons from the solution to reduce the silver atoms. This causes further attachment and reduction within a period of 30 s, resulting in the congregation of reduced silver ions upon the gold nanoparticles to cause size enhancement (approx. 5–10 μm in diameter) that provides a remarkable improvement in visibility (Figures 4B and 4C). By combining the above technology with specific mAb combination selected by antigen-capture ELISA, our LFIA provides superior visual detection of antigen-antibody complexes (Figure 4D).

The LFIA showed no cross-reactivity to related human coronaviruses or other respiratory viruses using simulated nasopharyngeal swab specimens, indicating its high specificity (Figure 4E). The detection limit of this LFIA was found to be approximately 6.25 pg/mL of recombinant NP (Figure 4F). Subsequent study with inactivated SARS-CoV-2 particles revealed that the lowest detection limit of virus copy number corresponded to 6.3×10^4 copies/mL (Figure 4G). This was tested in parallel experiments with commercially available LFIA kits, Abbott Panbio COVID-19 Ag Rapid Test, Roche SARS-CoV-2 Rapid Antibody Test, SD Biosensor Standard Q COVID-19 Ag, and Fujirebio Espline SARS-CoV-2, to reveal that our LFIA detected the lowest amount of SARS-CoV-2 compared to the above-mentioned commercial kits (Figure 4G). Furthermore, our LFIA could detect all the three major circulating mutants of SARS-CoV-2; 501Y.V1-3 (Figure 4H).

We next evaluated the performance of the developed LFIA using clinical specimens (nasopharyngeal swab) that had been diagnosed by RT-PCR. Our LFIA did not show false positives (n = 63), revealing that the specificity was 100% (Figure 4I). Comparison experiments using nasopharyngeal swab specimens (n = 90) revealed that other antigen detection kits did not show false positives too. Nevertheless, none had a sensitivity as high as the LFIA developed in this study (Figure 4I). Notably, our LFIA performed better when compared to other antigen-detection kits in samples with intermediate viral load Ct values

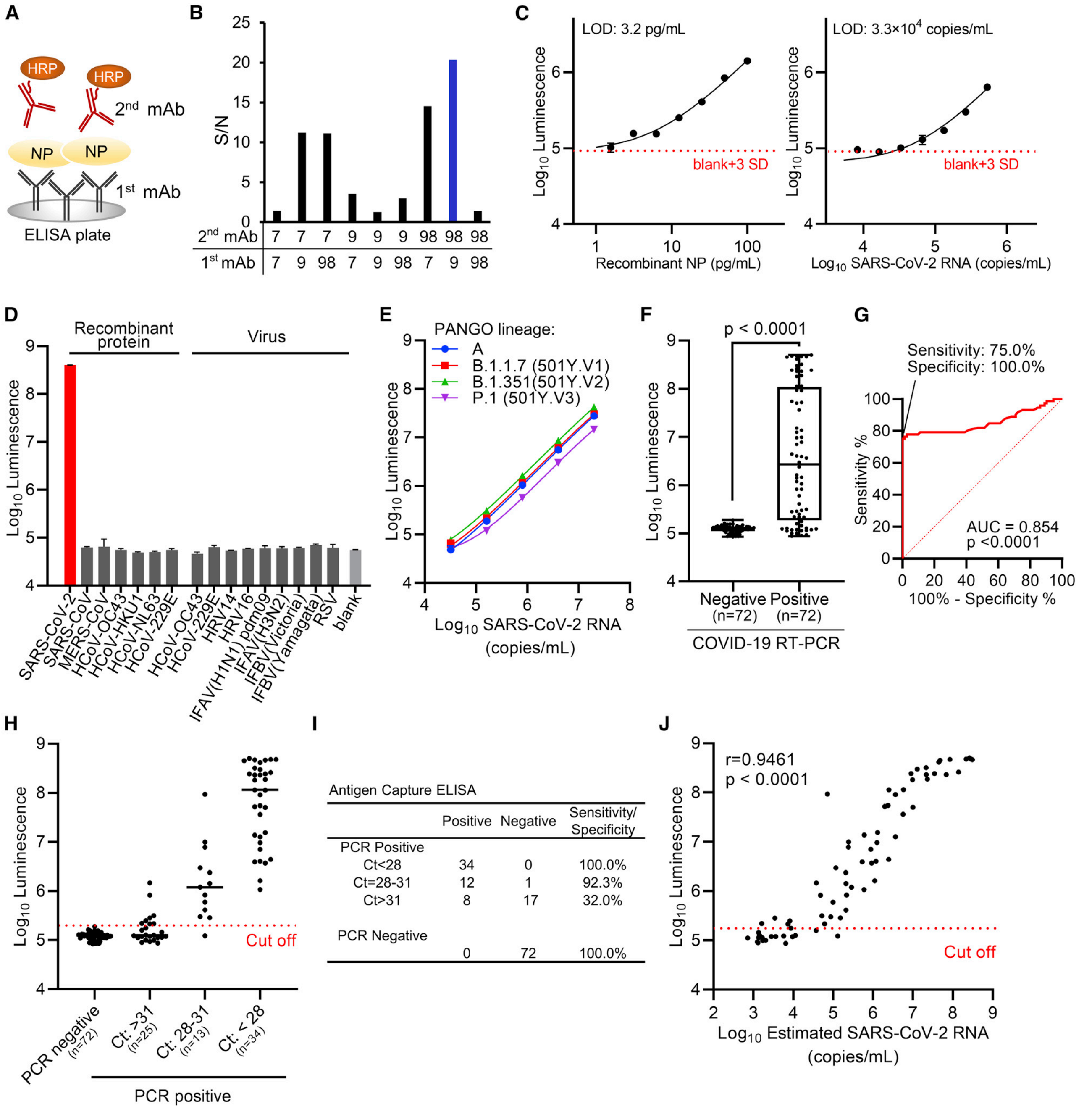

Antigen Capture ELISA	Positive	Negative	Sensitivity/ Specificity
PCR Positive			
Ct<28	34	0	100.0%
Ct=28-31	12	1	92.3%
Ct>31	8	17	32.0%
PCR Negative			
	0	72	100.0%

Figure 3. Development of antigen-capture ELISA

(A) Schematic representation of sandwich ELISA. Each mAb was labeled with horseradish peroxidase (HRP) and subjected to ELISA analysis. 9 pairs of antibodies were tested.

(B) Determination of the optimal combination of capturing and detection mAbs. S/N ratios for antigen detection by each of the 9 combinations were calculated in the presence of 2 ng/mL antigen versus blank (two technical replicates).

(C) Detection limit of ELISA. Serially diluted recombinant NP and inactivated SARS-CoV-2 were subjected to ELISA. Graph data are presented as mean ± SD (six technical replicates). The error bars represent SD. The detection limits of both recombinant NP protein and SARS-CoV-2 were determined according to the cutoff value, which was calculated by the formula (average +3SD).

(D) Specificity of ELISA. Simulated specimens positive for indicated viruses prepared by adding recombinant protein or common respiratory viruses to pooled COVID-19 RT-PCR-negative specimens were analyzed by ELISA. "Virus" indicates inactivated virus (at least 10^6 copies/mL for HCoV-229E and HCoV-OC43 or at least 10^5 $TCID_{50}$/mL [50% tissue culture infectious dose] for other viruses). "Recombinant protein" indicates recombinant NP antigen of human coronavirus (200 ng/mL). Graph data are presented as mean ± SD (three technical replicates). HRV, human rhinovirus; IFAV, influenza A virus; IFBV, influenza B virus; RSV, respiratory syncytial virus.

(legend continued on next page)

(Ct = 28–31) and low viral load Ct values (Ct > 31; Figure 4I). These results indicate that the LFIA designed with our newly developed mAbs could be useful for rapid and efficient detection of SARS-CoV-2 antigen.

DISCUSSION

Nearing a year since the outbreak, the COVID-19 pandemic continues to spread unchecked, causing serious health concerns worldwide. Apart from finding specific antiviral agents and vaccines, establishing a test for immediate detection of the infected and screening mass populations to initiate appropriate quarantine measures is necessary to control the spread of infection. The World Health Organization stresses that diagnostic testing for COVID-19 is critical to track the virus, understand its epidemiology to inform case management, and suppress transmission.[28] As of now, RT-PCR is the primary test available for establishing the diagnosis of COVID-19. Although NAT is sensitive and accurate, it requires specific equipment and trained personnel in laboratories with centralized services and takes a few hours to obtain results. Owing to their simplicity and ease of use, LFIA-based antigen detection kits can serve as POCT that can provide immediate results for prompt therapeutic or prophylactic actions. These advantages warrant the need for a reliable antigen detection kit that can supplement or supplant molecular diagnostics. To date, several antigen detection kits for SARS-CoV-2 are commercially available; however, almost all of them face the problem of cross-reactivity to other human coronaviruses, not revealing epitopes or specificities of antibodies, and may not be able to respond to forthcoming viral mutations.[8,29]

In the current study, we have developed robust antigen-detection systems using newly synthesized high-affinity mAbs that specifically target SARS-CoV-2 devoid of cross-reactivity with other human coronaviruses, including SARS-CoV. While designing an antigen detection system, NP appears to be a good choice as the target antigen because it is abundantly expressed in replicating virions and is highly immunogenic. Due to the above-mentioned reasons, NP is widely being used in serological diagnostic tests.[14] However, due to its high homology with other human coronaviruses, mAbs generated with whole NP of SARS-CoV-2 may potentially cross-react with antigens of other human coronaviruses, causing false-positive reactions in non-COVID-19 cases.[30–32] To overcome the problem of cross-reactivity, we produced ΔN-NP (aas 121–419) by deleting the conserved homologous aa sequences at the NTD and used this antigen to generate mAbs specific for SARS-CoV-2. We successfully created three specific mAbs that exclusively react with SARS-CoV-2 NP and not with any of the other related human coronaviruses.

Preparation of high-quality antigen is the most critical prerequisite for creating specific mAbs. Synthetic peptides containing predicted viral immunogenic epitopes are widely used to immunize animals for commercial generation of mAbs.[33,34] However, synthetic peptides are often linear and therefore are not ideally representative of the native features of antigens with regard to their actual spatial structures. Indeed, a previous study has demonstrated that the anti-SARS-CoV-2 NP mAbs created with the antigenic peptides were found to cross-react with other coronaviruses.[35] Also, the study showed that the sensitivity of the antigen-detection ELISA designed with antigenic peptide-derived mAbs was not so high (~100 ng/mL).[35] To overcome these problems, we synthesized the SARS-CoV-2 ΔN-NP with the wheat germ cell-free system. This system makes use of a eukaryotic translation machinery to synthesize properly folded and biologically active proteins structurally identical to those expressed in mammalian cells.[18,19] Although the antigen we used in this study was N-terminally deficient, it is highly likely to have a multimeric structure because of the presence of an oligomer-forming domain at the C-terminal dimerization domain.[36,37] Because the epitopes for our newly developed mAbs were exposed on the surface of the dimerized NP proteins, our antibodies were able to bind with native viral antigens. Yet another salient feature of these mAbs was that they detected the three major variants of concern (501Y.V1, 501Y.V2, and 501Y.V3) because these rampantly circulating mutants do not possess any mutations in the epitope-binding sites of our mAbs. These characteristics highlight the practical applicability of our mAbs for developing rapid antigen detection assays for COVID-19.

The overall sensitivity of our antigen-detection ELISA was 75.0%. However, we observed that the sensitivity increased with lower Ct values, and a similar trend was noticed even with the LFIA. Bullard et al.[38] reveal that viable and transmissible SARS-CoV-2 virions are present only in respiratory samples with Ct values <24 although higher Ct values comprise non-infectious genetic material. Both our ELISA and LFIA achieved 100% sensitivity when tested on PCR-positive clinical specimens with Ct values <28, suggesting that they might not miss out the detection of viable and transmissible viruses. However, further viral culture analysis is required to confirm this. The LFIA developed in our study differs from its conventional counterparts by possessing output signal amplification using silver halide photography technology.[26] Output signal-amplification techniques augment the performance of LFIAs by increasing their sensitivity.[39,40]

Our antigen-detection assays can be expected to perform as a clinical diagnostic tool. By detecting cultured SARS-CoV-2 particles as low as 6.3×10^4 copies/mL, our LFIA was more sensitive than other commercially available antigen-detection kits.

(E) Detection of variant of concerns strains by ELISA. All the three major variants were detected efficiently. Graph data are presented as mean ± SD (three technical replicates).
(F) Clinical performance of ELISA assay for nasal swab samples from RT-PCR negative (n = 72) and positive (n = 72). Boxplots of index values at ELISA assay were depicted. The p value was calculated using Welch's t test (two-tailed).
(G) Receiver operating characteristic curves for antigen-capture ELISA.
(H and I) Sensitivity and specificity of ELISA results according to real-time PCR cycle threshold (Ct) values group based on NIID-N2 primer set.
(J) Relation between RNA copy number in PCR-positive specimens and reactivity of antigen-capture ELISA (n = 72; Spearman's correlation).

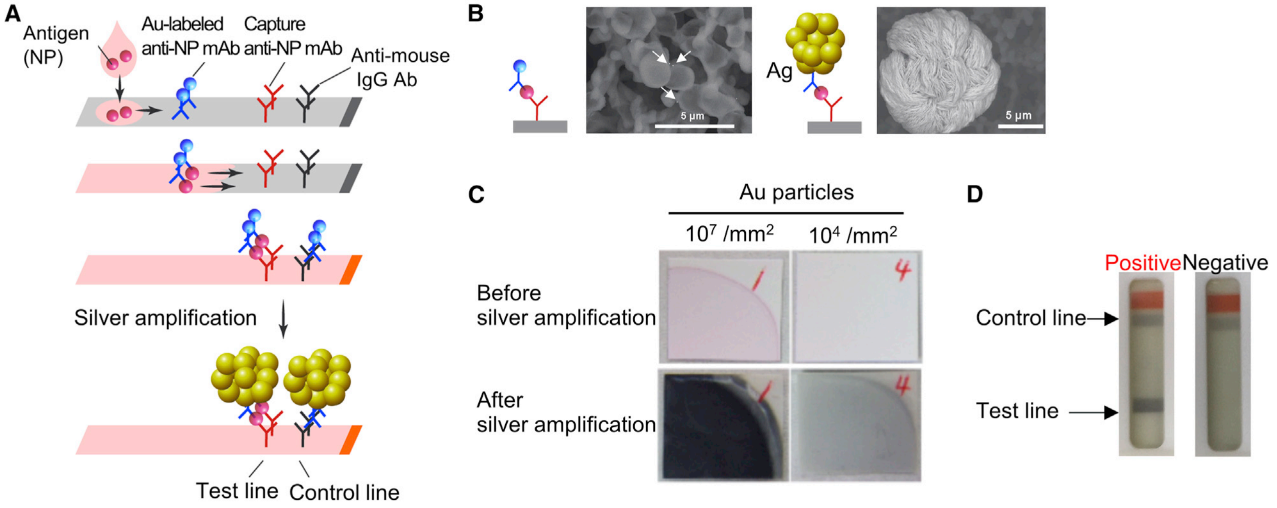

E

	Virus	rProtein
SARS-CoV-2	+	+
SARS-CoV	NT	–
MERS-CoV	NT	–
HCoV-OC43	–	–
HCoV-HKU1	NT	–
HCoV-NL63	NT	–
HCoV-229E	–	–
HRV14	–	NT
HRV16	–	NT
IFAV(H1N1) pdm09	–	NT
IFAV(H3N2)	–	NT
IFBV(Victoria)	–	NT
IFBV(Yamagata)	–	NT
RSV	–	NT

F

rNP (pg/mL)	50	25	12.5	6.25	3.13	1.56	0
This study	+	+	+	+	–	–	–

G

SARS-CoV-2 (copies/mL)	2.0×10^8	2.0×10^6	1.0×10^6	5.0×10^5	2.5×10^5	1.3×10^5	6.3×10^4	3.1×10^4
This study	+	+	+	+	+	+	+	–
Abbott	+	+	–	–	–	–	–	–
Roche	+	+	+	–	–	–	–	–
SD Biosensor	+	+	–	–	–	–	–	–
Fujirebio	+	+	+	+	–	–	–	–

H

SARS-CoV-2 PANGO lineage	Virus	rProtein
A	+	+
B.1.1.7 (501Y.V1)	+	+
B.1.351 (501Y.V2)	+	+
P.1 (501Y.V3)	+	+
blank	–	–

I

This study

	Positive	Negative	Sensitivity/ Specificity
PCR Positive			
Ct<28	17	0	100.0%
Ct=28-31	15	1	93.8%
Ct>31	5	7	41.7%
PCR Negative	0	63	100.0%

Abbott

	Positive	Negative	Sensitivity/ Specificity
PCR Positive			
Ct<28	17	0	100.0%
Ct=28-31	11	5	68.8%
Ct>31	2	10	16.7%
PCR Negative	0	45	100.0%

Roche

	Positive	Negative	Sensitivity/ Specificity
PCR Positive			
Ct<28	17	0	100.0%
Ct=28-31	15	1	93.8%
Ct>31	3	9	25.0%
PCR Negative	0	45	100.0%

SD BIOSENSOR

	Positive	Negative	Sensitivity/ Specificity
PCR Positive			
Ct<28	17	0	100.0%
Ct=28-31	15	1	93.8%
Ct>31	3	9	25.0%
PCR Negative	0	45	100.0%

FUJIREBIO

	Positive	Negative	Sensitivity/ Specificity
PCR Positive			
Ct<28	17	0	100.0%
Ct=28-31	15	1	93.8%
Ct>31	1	11	8.3%
PCR Negative	0	45	100.0%

Figure 4. Immunochromatographic test using developed monoclonal antibodies with silver amplification technology

(A) Schematic diagram of lateral flow immunoassay with silver amplification technology. The antigen in the sample dropped into the device flows on the cellulose membrane together with the colloidal gold-labeled anti-SARS-CoV-2 NP antibody, and when captured by the membrane-immobilized capture antibody, it develops color and appears as a single band. Adherence of silver ions to the surface of a catalytic gold nanoparticle causes electrons to reduce the silver atoms, leading to the size enhancement followed by 1,000-fold improvement in visibility.

(B and C) Size differences in SEM images (B) and naked eye visualized bands (C) with and without silver amplification.

(D) Representative test result for positive and negative.

(legend continued on next page)

Furthermore, our LFIA showed high sensitivity using clinical specimens, especially in samples with intermediate (Ct = 28–31) and low viral load (Ct > 31). On the other hand, there were slight discrepancies in the performance of each LFIA while detecting SARS-CoV-2 in culture supernatants and clinical specimens. For example, Fujirebio showed higher sensitivity than SD Biosensor in culture supernatants, although the latter performed better than the former in clinical specimens. This may be in part attributable to the property of clinical specimens, such as viscosity or presence of assay inhibitors. Further large-scale studies will be necessary to precisely identify the factors that may affect the performance of the assay in clinical samples, including saliva and sputum.

Like RT-PCR, antigen-detection tests also face the problem of handling infectious material, which can pose hazard to the handling persons. However, this could be overcome by inactivating the virus in the swab soon after collection by immersing into extracting solutions that contain surfactants. Previous reports have demonstrated that more than 0.1% of the non-ionic surfactant, such as NP-40 or Triton X-100, completely inactivates SARS-CoV-2.[41] We used extraction buffer containing non-ionic surfactant (0.5% for ELISA and 0.1% for LFIA), which did not cause any hindrance to the performance of the antigen-detection tests. Inactivation of SARS-CoV-2 was actually verified by the extraction buffer used in our study (data not shown).

Limitations of the study

Our study has a few limitations. We have found that Ala217 for no. 7, Asn345 for no. 9, and Gln390 for no. 98 were the critical residues to discriminate SARS-CoV-2 from SARS-CoV. Although our tests effectively distinguished both the viruses in clinical specimen, it is necessary to perform crystal structure analysis to confirm whether the epitope of the antibody is indeed present on the surface of NP. Our analysis did not include additional patient cohort for independent validation because we mainly dealt with the development of mAbs and their usage for antigen-detection assays. Clinical studies with a larger sample size through prospective trials are needed to validate our ELISA and LFIA before considering for clinical application. If this is done, our antigen-detection assays could possibly meet the urgent demand for rapid testing in clinical and community settings and support the unprecedented global fight against COVID-19.

STAR★METHODS

Detailed methods are provided in the online version of this paper and include the following:

- KEY RESOURCES TABLE
- RESOURCE AVAILABILITY
  - Lead contact
  - Materials availability
  - Data and code availability
- EXPERIMENTAL MODEL AND SUBJECT DETAILS
  - Clinical specimens and ethical statement
  - Cell culture
  - Preparation of viruses
- METHOD DETAILS
  - Expression plasmid
  - Cell-free protein synthesis and purification
  - Development of monoclonal antibodies
  - Immunoblot, immunostaining and immunohistochemical analysis
  - K_D determinations using Bio-Layer interferometry (BLI)
  - Epitope mapping of monoclonal antibodies
  - Homology modeling of SARS-CoV-2-NP and epitope localization analysis
  - Comparison of commercially available existing mAbs
  - Bioinformatic analysis
  - Sandwich ELISA
  - Lateral flow immunochromatography assay (LFIA)
  - Evaluation using clinical respiratory samples
  - Comparison of SARS-CoV-2 antigen detection kits
- QUANTIFICATION AND STATISTICAL ANALYSIS

SUPPLEMENTAL INFORMATION

Supplemental information can be found online at https://doi.org/10.1016/j.xcrm.2021.100311.

ACKNOWLEDGMENTS

We thank Drs. Naohito Nozaki, Satoko Matsunaga, Chizu Suzuki, Natsumi Takaira, Kenji Yoshihara, and Kazuo Horikawa for technical assistance. This work was supported in part by Japan Agency for Medical Research and Development (AMED) (grant numbers: JP19fk0108110 and JP20he0522001), by Health Labour Sciences research grant from The Ministry of Health Labour and Welfare (grant number: 19HA1003), and by collaborative research grant from Kanto Chemical Co., Inc. and FUJIFILM Corporation to A.R.

AUTHOR CONTRIBUTIONS

Y. Yamaoka and K.M. designed and performed the research, analyzed the data, and wrote the manuscript; S.S.J. analyzed the data and wrote the manuscript; R.F., K.O., S.K., J.K., A.W., T.T., M.N., T. Morita, H. Hayashi, H.M., K. Suzuki, D.A., and Y. Yoshimura performed the research and analyzed the data; K. Shimizu, H.O., S.U., C.K., N.T., T. Miyazaki, and E.Y. contributed the clinical specimen preparation; T.S. contributed the virus preparation; H.K., H.S., N.O., and H. Hasegawa analyzed the data; and A.R. directed the research, analyzed the data, and wrote the manuscript.

(E) Specificity of LFIA. Simulated specimens positive for indicated viruses prepared by adding recombinant protein or common respiratory viruses to pooled COVID-19 RT-PCR-negative specimens were analyzed by LFIA. Virus indicates inactivated virus (at least 10^6 copies/mL for HCoV-229E and HCoV-OC43 or at least 10^5 $TCID_{50}$/mL for other viruses). "Protein" indicates recombinant NP antigen of corresponding virus (200 ng/mL). NT, not tested (two technical replicates).
(F and G) Detection limit of LFIA. Recombinant protein (F) and inactivated SARS-CoV-2 (G) were subjected to LFIA analysis. Detection limit using SARS-CoV-2 was compared with indicated antigen detection kits. + and − indicate positive and negative detection, respectively (two technical replicates).
(H) Detection of variant strains of SARS-CoV-2. Virus indicates inactivated virus (less than 1.5×10^6 copies/mL). Protein indicates recombinant NP antigen from each strain (less than 100 pg/mL; two technical replicates).
(I) Sensitivity and specificity of indicated SARS-CoV-2 antigen-detection kits in PCR-positive (n = 45) and negative (n = 63 for this study; n = 45 for the others) specimens in nasopharyngeal swabs.

DECLARATION OF INTERESTS

Y. Yamaoka, S.K., K. Suzuki, and D.A. are current employees of Kanto Chemical Co., Inc.; J.K., A.W., and T.T. are current employees of FUJIFILM Corporation. A.R. received collaborative research grant from Kanto Chemical Co., Inc. and FUJIFILM Corporation. Provisional patent applications relevant to this study were filed. The remaining authors declare no competing interests.

Received: October 25, 2020
Revised: April 2, 2021
Accepted: May 13, 2021
Published: May 16, 2021

REFERENCES

1. Zhou, P., Yang, X.-L., Wang, X.-G., Hu, B., Zhang, L., Zhang, W., Si, H.-R., Zhu, Y., Li, B., Huang, C.-L., et al. (2020). A pneumonia outbreak associated with a new coronavirus of probable bat origin. Nature *579*, 270–273.
2. Durrheim, D.N., and Baker, M.G. (2020). COVID-19-a very visible pandemic. Lancet *396*, e17.
3. World Health Organization (2021). WHO coronavirus disease (COVID-19) dashboard. https://covid19.who.int/.
4. Scohy, A., Anantharajah, A., Bodéus, M., Kabamba-Mukadi, B., Verroken, A., and Rodriguez-Villalobos, H. (2020). Low performance of rapid antigen detection test as frontline testing for COVID-19 diagnosis. J. Clin. Virol. *129*, 104455.
5. Liu, J., Huang, J., and Xiang, D. (2020). Large SARS-CoV-2 outbreak caused by asymptomatic traveler, China. Emerg. Infect. Dis. *26*, 2260–2263.
6. Corman, V.M., Landt, O., Kaiser, M., Molenkamp, R., Meijer, A., Chu, D.K.W., Bleicker, T., Brünink, S., Schneider, J., Schmidt, M.L., et al. (2020). Detection of 2019 novel coronavirus (2019-nCoV) by real-time RT-PCR. Euro Surveill. *25*, 1–8.
7. Kitagawa, Y., Orihara, Y., Kawamura, R., Imai, K., Sakai, J., Tarumoto, N., Matsuoka, M., Takeuchi, S., Maesaki, S., and Maeda, T. (2020). Evaluation of rapid diagnosis of novel coronavirus disease (COVID-19) using loop-mediated isothermal amplification. J. Clin. Virol. *129*, 104446.
8. SD Biosensor (2020). COVID-19 Ag. http://sdbiosensor.com/xe/?module=file&act=procFileDownload&file_srl=23150&sid=9d51d887fee4e82c4b0a95c02c22a6c1&module_srl=513.
9. Quidel (2020). Technical bulletin of Sofia SARS antigen FIA.. https://www.quidel.com/sites/default/files/product/documents/TB10181000EN00.pdf.
10. Abbott Diagnostics (2020). BinaxNOW COVID-19 Ag CARD: instructions for use. US Food and Drug Administration website. https://www.fda.gov/media/141570/download.
11. Centers for Disease Control and Prevention (2021). SARS-CoV-2 variants of concern. https://www.cdc.gov/coronavirus/2019-ncov/cases-updates/variant-surveillance/variant-info.html.
12. Food and Drug Administration (2021). Genetic variants of SARS-CoV-2 may lead to false negative results with molecular tests for detection of SARS-CoV-2 - Letter to clinical laboratory staff and health care providers. https://www.fda.gov/medical-devices/letters-health-care-providers/genetic-variants-sars-cov-2-may-lead-false-negative-results-molecular-tests-detection-sars-cov-2.
13. Kogaki, H., Uchida, Y., Fujii, N., Kurano, Y., Miyake, K., Kido, Y., Kariwa, H., Takashima, I., Tamashiro, H., Ling, A.E., and Okada, M. (2005). Novel rapid immunochromatographic test based on an enzyme immunoassay for detecting nucleocapsid antigen in SARS-associated coronavirus. J. Clin. Lab. Anal. *19*, 150–159.
14. Dutta, N.K., Mazumdar, K., and Gordy, J.T. (2020). The nucleocapsid protein of SARS-CoV-2: a target for vaccine development. J. Virol. *94*, 1–2.
15. Chen, H., Cui, Y., Han, X., Hu, W., Sun, M., Zhang, Y., Wang, P.-H., Song, G., Chen, W., and Lou, J. (2020). Liquid-liquid phase separation by SARS-CoV-2 nucleocapsid protein and RNA. Cell Res. *30*, 1143–1145.
16. Takeda, H., Ogasawara, T., Ozawa, T., Muraguchi, A., Jih, P.J., Morishita, R., Uchigashima, M., Watanabe, M., Fujimoto, T., Iwasaki, T., et al. (2015). Production of monoclonal antibodies against GPCR using cell-free synthesized GPCR antigen and biotinylated liposome-based interaction assay. Sci. Rep. *5*, 11333.
17. Senchi, K., Matsunaga, S., Hasegawa, H., Kimura, H., and Ryo, A. (2013). Development of oligomannose-coated liposome-based nasal vaccine against human parainfluenza virus type 3. Front. Microbiol. *4*, 346.
18. Endo, Y., and Sawasaki, T. (2006). Cell-free expression systems for eukaryotic protein production. Curr. Opin. Biotechnol. *17*, 373–380.
19. Takai, K., and Endo, Y. (2010). The cell-free protein synthesis system from wheat germ. Methods Mol. Biol. *607*, 23–30.
20. Goshima, N., Kawamura, Y., Fukumoto, A., Miura, A., Honma, R., Satoh, R., Wakamatsu, A., Yamamoto, J., Kimura, K., Nishikawa, T., et al. (2008). Human protein factory for converting the transcriptome into an in vitro-expressed proteome. Nat. Methods *5*, 1011–1017.
21. Yamaoka, Y., Matsuyama, S., Fukushi, S., Matsunaga, S., Matsushima, Y., Kuroyama, H., Kimura, H., Takeda, M., Chimuro, T., and Ryo, A. (2016). Development of monoclonal antibody and diagnostic test for Middle East respiratory syndrome coronavirus using cell-free synthesized nucleocapsid antigen. Front. Microbiol. *7*, 509.
22. Yamaoka, Y., Jeremiah, S.S., Miyakawa, K., Saji, R., Nishii, M., Takeuchi, I., and Ryo, A. (2021). Whole nucleocapsid protein of severe acute respiratory syndrome coronavirus 2 may cause false-positive results in serological assays. Clin. Infect. Dis. *72*, 1291–1292.
23. Kang, S., Yang, M., Hong, Z., Zhang, L., Huang, Z., Chen, X., He, S., Zhou, Z., Zhou, Z., Chen, Q., et al. (2020). Crystal structure of SARS-CoV-2 nucleocapsid protein RNA binding domain reveals potential unique drug targeting sites. Acta Pharm. Sin. B *10*, 1228–1238.
24. Zinzula, L., Basquin, J., Bohn, S., Beck, F., Klumpe, S., Pfeifer, G., Nagy, I., Bracher, A., Hartl, F.U., and Baumeister, W. (2021). High-resolution structure and biophysical characterization of the nucleocapsid phosphoprotein dimerization domain from the Covid-19 severe acute respiratory syndrome coronavirus 2. Biochem. Biophys. Res. Commun. *538*, 54–62.
25. Wu, S., Tian, C., Liu, P., Guo, D., Zheng, W., Huang, X., Zhang, Y., and Liu, L. (2021). Effects of SARS-CoV-2 mutations on protein structures and intraviral protein-protein interactions. J. Med. Virol. *93*, 2132–2140.
26. Broger, T., Sossen, B., du Toit, E., Kerkhoff, A.D., Schutz, C., Ivanova Reipold, E., Ward, A., Barr, D.A., Macé, A., Trollip, A., et al. (2019). Novel lipoarabinomannan point-of-care tuberculosis test for people with HIV: a diagnostic accuracy study. Lancet Infect. Dis. *19*, 852–861.
27. Wada, A., Sakoda, Y., Oyamada, T., and Kida, H. (2011). Development of a highly sensitive immunochromatographic detection kit for H5 influenza virus hemagglutinin using silver amplification. J. Virol. Methods *178*, 82–86.
28. World Health Organization (2020). Laboratory testing for coronavirus disease 2019 (COVID-19) in suspected human cases: interim guidance, 2 March 2020. https://apps.who.int/iris/handle/10665/331329.
29. Lambert-Niclot, S., Cuffel, A., Le Pape, S., Vauloup-Fellous, C., Morand-Joubert, L., Roque-Afonso, A.M., Le Goff, J., and Delaugerre, C. (2020). Evaluation of a rapid diagnostic assay for detection of sars-cov-2 antigen in nasopharyngeal swabs. J. Clin. Microbiol. *58*, e00977-20.
30. Yu, F., Le, M.Q., Inoue, S., Thai, H.T.C., Hasebe, F., Del Carmen Parquet, M., and Morita, K. (2005). Evaluation of inapparent nosocomial severe acute respiratory syndrome coronavirus infection in Vietnam by use of highly specific recombinant truncated nucleocapsid protein-based enzyme-linked immunosorbent assay. Clin. Diagn. Lab. Immunol. *12*, 848–854.
31. Khan, S., Nakajima, R., Jain, A., de Assis, R.R., Jasinskas, A., Obiero, J., Adenaiye, O., Tai, S., Hong, F., Milton, D., et al. (2020). Analysis of serologic cross-reactivity between common human coronaviruses and SARS-CoV-2 using coronavirus antigen microarray. bioRxiv. https://doi.org/10.1101/2020.03.24.006544.

CellPress
OPEN ACCESS

32. Rosadas, C., Randell, P., Khan, M., McClure, M.O., and Tedder, R.S. (2020). Testing for responses to the wrong SARS-CoV-2 antigen? Lancet *396*, e23.

33. Zvirbliene, A., Samonskyte, L., Gedvilaite, A., Voronkova, T., Ulrich, R., and Sasnauskas, K. (2006). Generation of monoclonal antibodies of desired specificity using chimeric polyomavirus-derived virus-like particles. J. Immunol. Methods *311*, 57–70.

34. Song, D., Ha, G., Serhan, W., Eltahir, Y., Yusof, M., Hashem, F., Elsayed, E., Marzoug, B., Abdelazim, A., and Al Muhairi, S. (2015). Development and validation of a rapid immunochromatographic assay for detection of Middle East respiratory syndrome coronavirus antigen in dromedary camels. J. Clin. Microbiol. *53*, 1178–1182.

35. Li, M., Jin, R., Peng, Y., Wang, C., Ren, W., Lv, F., Gong, S., Fang, F., Wang, Q., Li, J., et al. (2020). Generation of antibodies against COVID-19 virus for development of diagnostic tools. medRxiv. https://doi.org/10.1101/2020.02.20.20025999.

36. Zhou, R., Zeng, R., von Brunn, A., and Lei, J. (2020). Structural characterization of the C-terminal domain of SARS-CoV-2 nucleocapsid protein. Mol. Biomed. *1*, 2.

37. Ye, Q., West, A.M.V., Silletti, S., and Corbett, K.D. (2020). Architecture and self-assembly of the SARS-CoV-2 nucleocapsid protein. Protein Sci. *29*, 1890–1901.

38. Bullard, J., Dust, K., Funk, D., Strong, J.E., Alexander, D., Garnett, L., Boodman, C., Bello, A., Hedley, A., Schiffman, Z., et al. (2020). Predicting infectious severe acute respiratory syndrome coronavirus 2 from diagnostic samples. Clin. Infect. Dis. *71*, 2663–2666.

39. Sakurai, A., Takayama, K., Nomura, N., Kajiwara, N., Okamatsu, M., Yamamoto, N., Tamura, T., Yamada, J., Hashimoto, M., Sakoda, Y., et al. (2015). Fluorescent immunochromatography for rapid and sensitive typing of seasonal influenza viruses. PLoS ONE *10*, e0116715.

40. Morioka, K., Urayama, K., Wada, A., Yoshida, K., Kato, T., Kitano, R., Nishi, T., Kanno, T., Yamada, M., Yamakawa, M., et al. (2020). Development and evaluation of silver amplification immunochromatography kit for foot-and-mouth disease virus antigen detection. J. Virol. Methods *275*, 113736.

41. Welch, S.R., Davies, K.A., Buczkowski, H., Hettiarachchi, N., Green, N., Arnold, U., Jones, M., Hannah, M.J., Evans, R., Burton, C., et al. (2020). Analysis of inactivation of SARS-CoV-2 by specimen transport media, nucleic acid extraction reagents, detergents, and fixatives. J. Clin. Microbiol. *58*, e01713-20.

42. Shirato, K., Nao, N., Katano, H., Takayama, I., Saito, S., Kato, F., Katoh, H., Sakata, M., Nakatsu, Y., Mori, Y., et al. (2020). Development of genetic diagnostic methods for detection for novel coronavirus 2019(nCoV-2019) in Japan. Jpn. J. Infect. Dis. *73*, 304–307.

43. Vijgen, L., Keyaerts, E., Moës, E., Maes, P., Duson, G., and Van Ranst, M. (2005). Development of one-step, real-time, quantitative reverse transcriptase PCR assays for absolute quantitation of human coronaviruses OC43 and 229E. J. Clin. Microbiol. *43*, 5452–5456.

44. Webb, B., and Sali, A. (2014). Protein structure modeling with MODELLER. Methods Mol. Biol. *1137*, 1–15.

45. Guex, N., and Peitsch, M.C. (1997). SWISS-MODEL and the Swiss-PdbViewer: an environment for comparative protein modeling. Electrophoresis *18*, 2714–2723.

46. Pettersen, E.F., Goddard, T.D., Huang, C.C., Couch, G.S., Greenblatt, D.M., Meng, E.C., and Ferrin, T.E. (2004). UCSF Chimera–a visualization system for exploratory research and analysis. J. Comput. Chem. *25*, 1605–1612.

47. Katoh, K., Rozewicki, J., and Yamada, K.D. (2019). MAFFT online service: multiple sequence alignment, interactive sequence choice and visualization. Brief. Bioinform. *20*, 1160–1166.

48. Edgar, R.C. (2004). MUSCLE: multiple sequence alignment with high accuracy and high throughput. Nucleic Acids Res. *32*, 1792–1797.

49. Kumar, S., Stecher, G., Li, M., Knyaz, C., and Tamura, K. (2018). MEGA X: Molecular Evolutionary Genetics Analysis across Computing Platforms. Mol. Biol. Evol. *35*, 1547–1549.

50. Matsuyama, S., Nao, N., Shirato, K., Kawase, M., Saito, S., Takayama, I., Nagata, N., Sekizuka, T., Katoh, H., Kato, F., et al. (2020). Enhanced isolation of SARS-CoV-2 by TMPRSS2-expressing cells. Proc. Natl. Acad. Sci. USA *117*, 7001–7003.

51. Kawakami, C., Yamayoshi, S., Akimoto, M., Nakamura, K., Miura, H., Fujisaki, S., Pattinson, D.J., Shimizu, K., Ozawa, H., Momoki, T., et al. (2019). Genetic and antigenic characterisation of influenza A(H3N2) viruses isolated in Yokohama during the 2016/17 and 2017/18 influenza seasons. Euro Surveill. *24*, 1–9.

52. Matsunaga, S., Kawakami, S., Matsuo, I., Okayama, A., Tsukagoshi, H., Kudoh, A., Matsushima, Y., Shimizu, H., Okabe, N., Hirano, H., et al. (2014). Wheat germ cell-free system-based production of hemagglutinin-neuraminidase glycoprotein of human parainfluenza virus type 3 for generation and characterization of monoclonal antibody. Front. Microbiol. *5*, 208.

53. Kimura, K., Nozaki, N., Saijo, M., Kikuchi, A., Ui, M., and Enomoto, T. (1994). Identification of the nature of modification that causes the shift of DNA topoisomerase II β to apparent higher molecular weight forms in the M phase. J. Biol. Chem. *269*, 24523–24526.

54. Kimura, K., Nozaki, N., Enomoto, T., Tanaka, M., and Kikuchi, A. (1996). Analysis of M phase-specific phosphorylation of DNA topoisomerase II. J. Biol. Chem. *271*, 21439–21445.

55. Yang, J., Yan, R., Roy, A., Xu, D., Poisson, J., and Zhang, Y. (2015). The I-TASSER Suite: protein structure and function prediction. Nat. Methods *12*, 7–8.

56. Yang, O.O. (2009). Candidate vaccine sequences to represent intra- and inter-clade HIV-1 variation. PLoS ONE *4*, e7388.

STAR★METHODS

KEY RESOURCES TABLE

REAGENT or RESOURCE	SOURCE	IDENTIFIER
Antibodies		
Anti-SARS-CoV-2 NP mAb #7	This study	N/A
Anti-SARS-CoV-2 NP mAb #9	This study	N/A
Anti-SARS-CoV-2 NP mAb #98	This study	N/A
Anti-His-tag mAb	MEDICAL & BIOLOGICAL LABORATORIES CO., LTD.	Cat# D291-3；RRID: AB_10597733
Anti-DDDDK-tag (Flag) mAb	MEDICAL & BIOLOGICAL LABORATORIES CO., LTD.	Cat#M185-3L; RRID: AB_11123930
SARS-CoV-2 Nucleocapsid Protein Monoclonal Antibody (7E1B)	Bioss Antibodies Inc.	Cat#bsm-41414M; RRID: AB_2848129
SARS coronavirus monoclonal antibody	BiosPacific, Inc.	Cat#A03070041P
SARS coronavirus monoclonal antibody	BiosPacific, Inc.	Cat#A03080041P
Anti-Mouse IgG, HRP-Linked F(ab')$_2$ Fragment Sheep	Cytiva	Cat#NA9310-1ML; RRID: AB_772193
Goat anti-Mouse IgG (H+L) Highly Cross-Adsorbed Secondary Antibody, Alexa Fluor 568	Thermo Fisher Scientific	Cat#A-11031; RRID: AB_144696
Bacterial and virus strains		
SARS-CoV-2 isolate (2019-nCoV/JPN/TY/WK-521/2020)	National Institute of Infectious Diseases, Japan	N/A
SARS-CoV-2 isolate (2019-nCoV/JPN/QHN001/2021)	National Institute of Infectious Diseases, Japan	N/A
SARS-CoV-2 isolate (2019-nCoV/JPN/TY8-612/2021)	National Institute of Infectious Diseases, Japan	N/A
SARS-CoV-2 isolate (2019-nCoV/JPN/TY7-501/2021)	National Institute of Infectious Diseases, Japan	N/A
Human coronavirus OC43	American Type Culture Collection (ATCC)	Cat#VR-1558
Human coronavirus 229E	American Type Culture Collection (ATCC)	Cat#VR-740
Human rhino virus 14 (HRV14)	American Type Culture Collection (ATCC)	Cat#VR-284
Human rhino virus 14 (HRV16)	American Type Culture Collection (ATCC)	Cat#VR-283
Respiratory syncytial virus (RSV)	American Type Culture Collection (ATCC)	Cat#VR-26
Influenza A virus (IFAV) H1N1 pdm09 isolate (A/Yokohama/72/2020)	Yokohama City Institute of Public Health, Kanagawa, Japan	N/A
Influenza A virus (IFAV) H3N2 isolate (A/Yokohama/68/2020)	Yokohama City Institute of Public Health, Kanagawa, Japan	N/A
Influenza B virus (IFBV) Victoria lineage isolate (B/Yokohama/33/2020)	Yokohama City Institute of Public Health, Kanagawa, Japan	N/A
Influenza B virus (IFBV) Yamagata lineage isolate (B/Yokohama/35/2019)	Yokohama City Institute of Public Health, Kanagawa, Japan	N/A
Chemicals, peptides, and recombinant proteins		
PrimeSTAR Mutagenesis Basal kit	Takara Bio Inc.	Cat#R046A
WEPRO7240H Expression Kit	CellFree Sciences Co.,Ltd.	Cat#CFS-TRI-7240H
WEPRO7240G Expression Kit	CellFree Sciences Co.,Ltd.	Cat#CFS-TRI-7240G
Hi-QRAS Gel N	Kanto Chemical Co., Inc.	Cat#49902-58
Rapid CBB KANTO 3S	Kanto Chemical Co., Inc.	Cat#36533-79
His-tagged N-terminal deleted mutant of NP (ΔN-NP; 121-419)	This study	N/A

(*Continued on next page*)

Continued

REAGENT or RESOURCE	SOURCE	IDENTIFIER
Ni-Sepharose Fast Flow beads	Cytiva	Cat#17531801
PreScission Protease	Cytiva	Cat#27084301
ABTS Microwell Peroxidase Substrate (2-Component System)	Kirkegaard & Perry Laboratories	Cat#5120-0032
TMB 1-Component Microwell Peroxidase Substrate, SureBlue	Kirkegaard & Perry Laboratories	Cat#5120-0075
SuperSignal West Femto Maximum Sensitivity Substrate	Thermo Fisher Scientific	Cat#34095
Dulbecco's modified Eagle's medium (high glucose)	FUJIFILM Wako Pure Chemical Corporation	Cat#044-29765
CD hybridoma medium AGT medium	Thermo Fisher Scientific	Cat#12372025
HYGM-7 Express (Ready - to - use), Liquid, without Phenol Red, protein free	Kanto Chemical Co., Inc.	Cat#49432-50
**Critical commercial assays**		
Standard Q COVID-19 Ag	SD. Biosensor	Cat#Q-NCOV-01G
Espline SARS-CoV-2	Fujirebio	Cat#260319
Panbio COVID-19 Ag Rapid Test	Abbott	Cat#4571226475003
SARS-CoV-2 Rapid Antigen Test	Roche	Cat#508487
**Deposited data**		
Partial structure of SARS-CoV-2 NP-1	Zinzula et al.[24]	PDB: 6YUN
Full-length structural model of MERS-NP	Yamaoka et al.[21]	N/A
Partial structure of SARS-CoV-2 NP-2	Kang et al.[23]	PDB: 6M3M
Full-length structural model of SARS-CoV-2 NP	This study	https://doi.org/10.17632/7b67yg29d6.1
Multiple sequence alignment of SARS-CoV-2 NP	This study	https://doi.org/10.17632/7b67yg29d6.1
**Experimental models: cell lines**		
HCT-8	American Type Culture Collection (ATCC)	CCL-244
MRC-5	RIKEN BioResource Research Center	RCB0211
VeroE6/TMPRSS2	Japanese Collection of Research Bioresources Cell Bank (JCRB)	JCRB1819
**Oligonucleotides**		
Reverse primer for the quantification of SARS-CoV-2: 5′- TGGCAGCTGT GTAGGTCAAC –3′	Shirato et al.[42]	N/A
Probe for the quantification of SARS-CoV-2: FAM-ATGTCGCGCATTGGCATGGA-BHQ	Shirato et al.[42]	N/A
Forward primer for the quantification of HCoV-229E: 5′- TTCCGACGT GCTCGAACTTT –3′	Vijgen et al.[43]	N/A
Reverse primer for the quantification of HCoV-229E: 5′- CCAACACGGTTG TGACAGTGA –3′	Vijgen et al.[43]	N/A
Probe for the quantification of HCoV-229E: FAM 5′- TCCTGAGGTCAATGCA –3′ TAMRA	Vijgen et al.[43]	N/A
Forward primer for the quantification of HCoV-OC43: 5′- ATGTTAGGCCGATA ATTGAGGACTAT –3′	Vijgen et al.[43]	N/A

(*Continued on next page*)

Continued		
REAGENT or RESOURCE	SOURCE	IDENTIFIER
Reverse primer for the quantification of HCoV-OC43: 5′- AATGTAAAGA TGGCCGCGTATT –3′	Vijgen et al.[43]	N/A
Probe for the quantification of HCoV-OC43: FAM 5′- CATACTCTGACGGTCACAAT –3′ TAMRA	Vijgen et al.[43]	N/A
Forward primer for the quantification of SARS-CoV-2: 5′- AAATTTTGGGG ACCAGGAAC –3′	Shirato et al.[42]	N/A
Software and algorithms		
ForteBio data analysis software	Fortebio	https://www.sartorius.com/en/products/protein-analysis/octet-systems-software
MODELLER9.15	Webb et al.[44]	https://salilab.org/modeller/9.15/release.html
Swiss PDB viewer 4.1	Guex et al.[45]	https://spdbv.vital-it.ch/
UCSF Chimera software 1.13.1	Pettersen et al.[46]	https://www.cgl.ucsf.edu/chimera/
MAFFT version 7	Kato et al.[47]	https://mafft.cbrc.jp/alignment/server/
MEGAX (MUSCLE)	Edgar et al.[48], Kumar et al.[49]	https://www.megasoftware.net/
Graphpad Prism 8.43	Graphpad Software	https://www.graphpad.com/
Other		
Immunochromatographic assay with silver amplification technology	This study (FUJIFILM Corporation)	Cat#16696722

RESOURCE AVAILABILITY

Lead contact

Further information and requests for resources and reagents should be directed to, and will be fulfilled by the lead contact, Akihide Ryo (aryo@yokohama-cu.ac.jp).

Materials availability

Monoclonal antibodies established in this study are available from Yokohama City University and Kanto Chemical Co., Inc., under MTA. Antigen detection kit developed in this study is commercially available from FUJIFILM Corporation (FUJIFILM, cat# 16696722).

Data and code availability

Original/source data for multiple sequence alignment and structural model of SARS-CoV-2 NP in the paper have been deposited to Mendeley Data: https://doi.org/10.17632/7b67yg29d6.1.

EXPERIMENTAL MODEL AND SUBJECT DETAILS

Clinical specimens and ethical statement

Clinical specimens of nasopharyngeal swabs from 252 individuals with suspected SARS-CoV-2 infection regardless of the onset of the disease, were retrieved for this study. These clinical specimens had been confirmed positive or negative for SARS-CoV-2 infection by RT-PCR prior using for evaluating the performance of antigen-capture ELISA and LFIA (72 positive and 72 negative specimens for antigen-capture ELISA, 45 positive and 63 negative specimens for LFIA). Demographic and descriptive information about the patients and healthy individuals such as the age and gender are not available due to ethical/privacy restriction. This study was approved by the Institutional Review Board of Yokohama City University (IRB No. B200800106), and the protocols used in the study were approved by the ethics committee.

Cell culture

VeroE6/TMPRSS2 cells (JCRB1819), HCT-8 cells (ATCC CCL-244), and MRC-5 cells (ATCC CCL-171) were cultured in Dulbecco's modified Eagle's medium (high glucose) (Wako, cat# 044-29765) supplemented with 10% (V/V) fetal bovine serum (FBS) and 1% penicillin-streptomycin in a 5% CO_2 at 37°C.

Preparation of viruses

SARS-CoV-2 clinical isolate (2019-nCoV/JPN/TY/WK-521/2020, 2019-nCoV/JPN/QHN001/2021, 2019-nCoV/JPN/TY8-612/2021, 2019-nCoV/JPN/TY7-501/2021: obtained from National Institute of Infectious Diseases, Japan) were propagated in Vero-E6 cells expressing TMPRSS2 (JCRB1819) as described previously[50]. The viral samples were inactivated by addition of NP-40 to a final concentration of 0.5% (v/v) prior to each immunoassay. Quantitation of viral copy number was carried out by RT-PCR using N2 primers and probe sets[42]. Human coronavirus OC43 (ATCC VR-1558) and 229E (ATCC VR-740) obtained from ATCC were propagated in HCT-8 cells (ATCC CCL-244) and MRC-5 cells (ATCC CCL-171), respectively, and viral copy number was quantified by RT-PCR as previously described[43]. Human rhinovirus (HRV) 14 (ATCC VR-284), HRV16 (ATCC VR-283), and Respiratory syncytial virus (RSV, ATCC VR-26) were propagated in MRC-5 cells and quantified by 50% Tissue Culture Infectious Dose ($TCID_{50}$) assay. Influenza A virus (IFAV) H1N1 pdm09 isolate (A/Yokohama/72/2020), IFAV H3N2 isolate (A/Yokohama/68/2020), Influenza B virus (IFBV) Victoria lineage isolate (B/Yokohama/33/2020), and IFBV Yamagata lineage isolate (B/Yokohama/35/2019) were cultured and quantified as previously described[51]. Each viral culture were carried out in 5% CO_2 at 37°C for SARS-CoV-2, RSV, IFAV, and IFBV, at 33°C for OC43, 229E, HRV14, and HRV16.

METHOD DETAILS

Expression plasmid

Complementary DNAs encoding nucleocapsid proteins of human coronaviruses (SARS-CoV-2, GenBank No. NC_045512; SARS-CoV, GenBank No. NC_004718; MERS-CoV, GenBank No. NC_019843; HCoV-HKU1, GenBank No. NC_006577; HCoV-OC43, GenBank No. NC_005147; HCoV-229E, GenBank No. NC_002645; HCoV-NL63, GenBank No. NC_005831) were synthesized. Synthetic cDNAs were digested with *Xho*I and KpnI and inserted into pEU-E01-His-TEV-MCS, pEU-bls-s1-MCS, and pEU-Flag-GST-PS-MCS. Deletion and substitution mutants of SARS-CoV-2 for epitope mapping were generated using the PrimeSTAR Mutagenesis Basal kit (Takara Bio, Otsu, Japan).

Cell-free protein synthesis and purification

In vitro transcription and cell-free protein synthesis were performed as previously described[21,52]. For cell-free protein synthesis, WEPRO7240H and WEPRO7240G wheat germ extract (CellFree Sciences, Yokohama, Japan) was used in the bilayer translation method as previously described[52]. Synthesized proteins were confirmed by SDS-PAGE followed by CBB staining with Rapid CBB KANTO 3S (Kanto chemical, Tokyo, Japan) and immunoblotting.

His-tagged N-terminal deleted mutant of NP (ΔN-NP; 121-419) for immunization were synthesized by a bilayer dialysis method using the wheat cell-free system according to manufacturer instructions. The protein purification was carried out using Ni-Sepharose Fast Flow beads (Cytiva, Waukesha, WI, USA) as previously described[21]. Full-length NP were synthesized as FLAG-GST tagged proteins and purified by Glutathione Sepharose 4 Fast Flow and PreScission Protease (Cytiva).

Development of monoclonal antibodies

Immunization of BALB/c mice and generation of hybridomas producing anti-SARS-CoV-2-NP antibody were carried out as previously described[52–54]. For hybridoma screening, indirect ELISA was performed using full-length NP and DHFR (negative control). These proteins were diluted with PBS (1 μg/mL) and then immobilized to ELISA plate (Thermo Fisher Scientific, Rockford, IL, USA). After blocking with PBS containing 2% skim milk for 1h, diluted hybridoma supernatant (1:25) were added and incubated for 1h. After three washes with PBS-T, wells were incubated with 100 μL of diluted HRP-conjugated anti-mouse IgG antibody (1: 5,000) for 1h. After additional three washes with PBS-T, 100 μL of ABTS Substrate (Kirkegaard & Perry Laboratories) was added and incubated for 30 min. The absorbance at 405-490 nm was measured on GloMax Explorer plate reader (Promega, WI, USA), and the signal-to-noise ratio (S/N) was calculated.

The AlphaScreen assay was performed using 384-well ProxiPlates (PerkinElmer, Boston, MA, USA). Biotinylated full-length NP or DHFR (negative control) were incubated with a 20-fold dilution of hybridoma supernatant in 15 μL of binding mixture containing reaction buffer (100 mM Tris-HCl, pH 8.0, 1 mg/ml BSA, 0.01% Tween-20) at 26°C for 30 min. Then, 10 μL of the combined detection mixture containing 0.04 μL protein G-conjugated acceptor beads and 0.04 μL streptavidin-coated donor beads (AlphaScreen IgG detection kit, PerkinElmer) in reaction buffer were incubated at 26°C for 1 h. Antigen-antibody interactions were analyzed using an Envision microplate reader (PerkinElmer). For purification of antibodies, hybridoma cells were grown in CD hybridoma medium AGT medium (Thermo Fisher Scientific, Rockford, IL, USA) or HYGM 7 medium (Kanto chemical, Tokyo, Japan). Antibody purification was carried out as previously described[21].

Immunoblot, immunostaining and immunohistochemical analysis

Cell lysates samples in SDS sample buffer were loaded onto 10%–20% SDS-PAGE using Hi-QRAS Gel N (Kanto Chemical, Tokyo, Japan), the proteins were electrotransferred onto an Immobilon-P PVDF Transfer Membrane (Millipore, Bedford, MA, USA). Immunoblotting using anti-SARS-CoV-2 NP mAbs, anti-His monoclonal antibody, or anti-DDDDK (FLAG) tag antibody (MBL, Aichi, Japan) was performed as previously described[21]. For immunostaining analysis, cells were fixed with 4% paraformaldehyde and were permeabilized with PBS 0.5% Triton X-100 in PBS. After blocking with PBS containing 3% BSA, cells were stained with hybridoma

culture supernatants (1:20 dilution) and Alexa Fluor568-conjugated secondary antibodies (Thermo Fisher Scientific). Cells were mounted using VECTASHIELD mounting medium with DAPI (Vector laboratories, CA, USA). For immunohistochemical analysis, formalin-fixed and paraffin-embedded tissue sections from the autopsy lung were de-paraffinized and rehydrated. After blocking with 5% goat serum, the sections were incubated with developed mAb #98 (1:20 dilution). After washing, the sections were incubated with the linker and then the polymer peroxidase-conjugated anti-mouse IgG solution (EnVision™ FLEX+; DAKO, Ely, UK), and then visualized with 3,3-diaminobenzidine (DAKO) and counterstained with hematoxylin.

K_D determinations using Bio-Layer interferometry (BLI)

Kinetic properties were determined by Bio-Layer interferometry (BLI) using Octet RED96 instrument (ForteBio, USA). The operating temperature was maintained at 30°C in 96 well black microplates (Greiner Bio-One, Monroe, USA) which were agitated at 1,000 rpm. For antibody screening, Anti-mouse IgG Capture biosensor tips (AMC) were loaded with each culture supernatants (1:4 dilution) for 5 min in PBS containing 0.1% BSA and 0.02% Tween20. The association of recombinant ΔN-NP protein at concentrations of 300 nM was measured for 3 min, followed by a 5-min-long dissociation phase. All measurements were corrected for baseline drift by subtracting a reference well. Data were analyzed using a 1:1 binding model with local fitting algorithms in the ForteBio data analysis software. For precise affinity measurement using purified monoclonal antibodies, AMC were loaded with 15 μg/mL of each mAbs for 5 min. The association of recombinant Full-length NP protein at concentrations of 50, 25, 12.5, 6.25, 3.13, 1.56, and 0.781 nM was measured for 3 min, followed by a 5-min-long dissociation phase. All measurements were corrected for baseline drift by subtracting a reference well. Data were analyzed using a 1:1 binding model with global fitting algorithms in the ForteBio data analysis software.

Epitope mapping of monoclonal antibodies

Flag-GST tagged deletion and substitution mutants of SARS-CoV-2 NP were produced by the wheat germ cell-free system. Expressed proteins were diluted with PBS (1:400) and then immobilized to ELISA plate (Thermo Fisher Scientific, Rockford, IL, USA). Following blocking with PBS-T containing 3% skim milk for 1h, serially diluted monoclonal antibodies (3000, 600, 120, 24, 4.8, 0.96 ng/mL) were added and incubated for 1h. After three washes with PBS-T, wells were incubated with 100 μL of diluted HRP-conjugated anti-mouse IgG antibody (1:25,000) for 1h. After additional five washes with PBS-T, 100 μL of TMB 1-component Microwell Peroxidase Substrate, SureBlue (Kirkegaard & Perry Laboratories) was added and incubated for 15 min. The reaction was terminated by adding 50 μL of 2 M H_2SO_4, and absorbance at 450-620 nm was measured on a plate reader. (Bio-rad, CA, USA). Calculation of EC_{50} were performed by using GraphPad Prism 8 software. Mutants with an over 5-fold increase in EC_{50} compared to full-length NP were defined as negative.

Homology modeling of SARS-CoV-2-NP and epitope localization analysis

The dimer model of full-length SARS-CoV-2-NP was constructed by homology modeling based on the partial structure of SARS-CoV-2 NP (PDB: 6YUN, PDB: 6M3M)[23,24] and MERS-CoV NP[21] using the MODELLER9.15 software[44]. Protein structures not registered in PDB were estimated by the I-TASSER servers and used as templates for homology modeling[55]. Energy minimization of the generated model was carried out using Swiss PDB viewer 4.1[45]. Surface localization of each epitope was determined using the UCSF Chimera software[46].

Comparison of commercially available existing mAbs

Commercially available monoclonal antibodies (Bioss: Mouse Monoclonal 7E1B, Biospacific −1: SARS coronavirus monoclonal antibody -A03070041P, Biospacific −2: SARS coronavirus monoclonal antibody -A03080041P) were evaluated by Bio-Layer interferometry and specificity analysis.

Bioinformatic analysis

Homology of nucleocapsid proteins among human Coronaviruses was analyzed by multiple sequence alignment using the MUSCLE software[48]. Total 12,441 SARS-CoV-2 whole genome sequences were downloaded from NCBI as of August 19, 2020. Partially sequenced genomes, sequences with ambiguous sites, and 100% identical sequences were removed for further analysis. Consequently, 8,127 SARS-CoV-2 sequences were aligned using the MAFFT version 7 and MEGAX[47,49]. Shannon entropy score was calculated for each position in the protein alignment as previously described[56].

Sandwich ELISA

Each mAb was diluted in PBS to a concentration of 2.5 μg/mL, and then added to an ELISA plate (clear or black) (Thermo Fisher Scientific, Rockford, IL, USA). After blocking with 2% skim milk, 100 μL of antigen protein (2 ng/mL) diluted with PBS-T or blank (PBS-T alone) was added and incubated for 60 min at RT. After three washes with PBS-T, 100 μL of each mAb conjugated with horseradish peroxidase (HRP) was added into each well and incubated for 60 min at RT. After washing with PBS-T, 100 μL of ABTS substrate solution (Kirkegaard & Perry Laboratories, Washington, D.C., USA) or SuperSignal West Femto Maximum Sensitivity Substrate (Thermo Fisher Scientific, Rockford, IL, USA) was added and incubated. Absorbance at 410-490 nm or luminescence was measured on a GloMax Explorer plate reader (Promega, WI, USA), and the signal-to-noise ratio (S/N) was calculated.

Lateral flow immunochromatography assay (LFIA)

LFIA was developed over the LFIA designed in previous study[26,27]. Briefly, purified monoclonal antibodies and anti-mouse IgG (Jackson Immuno Research, PA, USA) were diluted in 50 mM Tris buffer) and dropped onto nitrocellulose membrane (ADVANTEC, Tokyo, Japan) to give the test and control line, respectively. The membrane was then dried at 40°C for 30 min to immobilize antibodies. A reagent pad including monoclonal antibodies conjugated with colloidal gold (British Biocell Intonational, Crumlin, UK) was placed upstream of test lines as sample application point. A test strip and two sealed pots containing a reducing reagent (containing 0.47 mol/l $Fe(NH_4)_2(SO_4)_2$) and a silver-ion reagent (0.31 mol/l $AgNO_3$) were placed in a cartridge (Figure S7).

Evaluation using clinical respiratory samples

Schematic experimental flow was shown in Figure S8. Swabs were collected in 3 mL of viral transport medium (Becton Dickinson, NJ, USA or Sugiyama-gen, Tokyo, Japan). RNA extraction and RT-PCR using NIID-N2 set primer were carried out as an administrative inspection[42]. Remaining clinical samples diagnosed as SARS-CoV-2 positive or negative were centrifuged at 4°C for 30 min and the supernatant were stored for −80°C until their use in this study. For antigen capture ELISA, samples (55 μL) were mixed with an equal volume of extraction buffer (PBS containing 0.2% BSA and 1% NP-40) and each 100 μL were subjected. For LFIA, samples (60 μL) were mixed with twice volume of extraction buffer (Tris buffer containing 0.1% non-ionic surfactant) and one drop were subjected. For quantification of Ct value and viral RNA copy number indicated in this study, remaining samples were diluted 2-fold (ELISA) or 3-fold (LFIA) with viral transport medium, and then viral RNA was isolated from 140 μL of diluted specimens by using QIAamp Viral RNA Mini Kit (QIAGEN, Düsseldorf, Germany). One step RT-PCR was performed using the CFX96 Touch Real-Time PCR Detection System (Bio-rad, CA, USA) and NIID-N2 set primer.

Comparison of SARS-CoV-2 antigen detection kits

Commercially available rapid antigen detection kit of SARS-CoV-2 (Abbott Panbio COVID-19 Ag Rapid Test, Roche SARS-CoV-2 Rapid Antigen Test, SD. Biosensor Standard Q COVID-19 Ag, and Fujirebio Espline SARS-CoV-2) were used according to the manufacturers' instructions. For evaluation of clinical samples, each of the specimens were diluted three times with each extraction buffer and then subjected. Test line interpretations were made by at least two independent observers within the stipulated time.

QUANTIFICATION AND STATISTICAL ANALYSIS

All statistical analyses were performed by using GraphPad Prism 8 software. Graph data are presented as mean ± SD. Statistical details of the analyses can be found in the figure legends.

Cell Reports Medicine, Volume *2*

Supplemental information

Highly specific monoclonal antibodies and epitope identification against SARS-CoV-2 nucleocapsid protein for antigen detection tests

Yutaro Yamaoka, Kei Miyakawa, Sundararaj Stanleyraj Jeremiah, Rikako Funabashi, Koji Okudela, Sayaka Kikuchi, Junichi Katada, Atsuhiko Wada, Toshiki Takei, Mayuko Nishi, Kohei Shimizu, Hiroki Ozawa, Shuzo Usuku, Chiharu Kawakami, Nobuko Tanaka, Takeshi Morita, Hiroyuki Hayashi, Hideaki Mitsui, Keita Suzuki, Daisuke Aizawa, Yukihiro Yoshimura, Tomoyuki Miyazaki, Etsuko Yamazaki, Tadaki Suzuki, Hirokazu Kimura, Hideaki Shimizu, Nobuhiko Okabe, Hideki Hasegawa, and Akihide Ryo

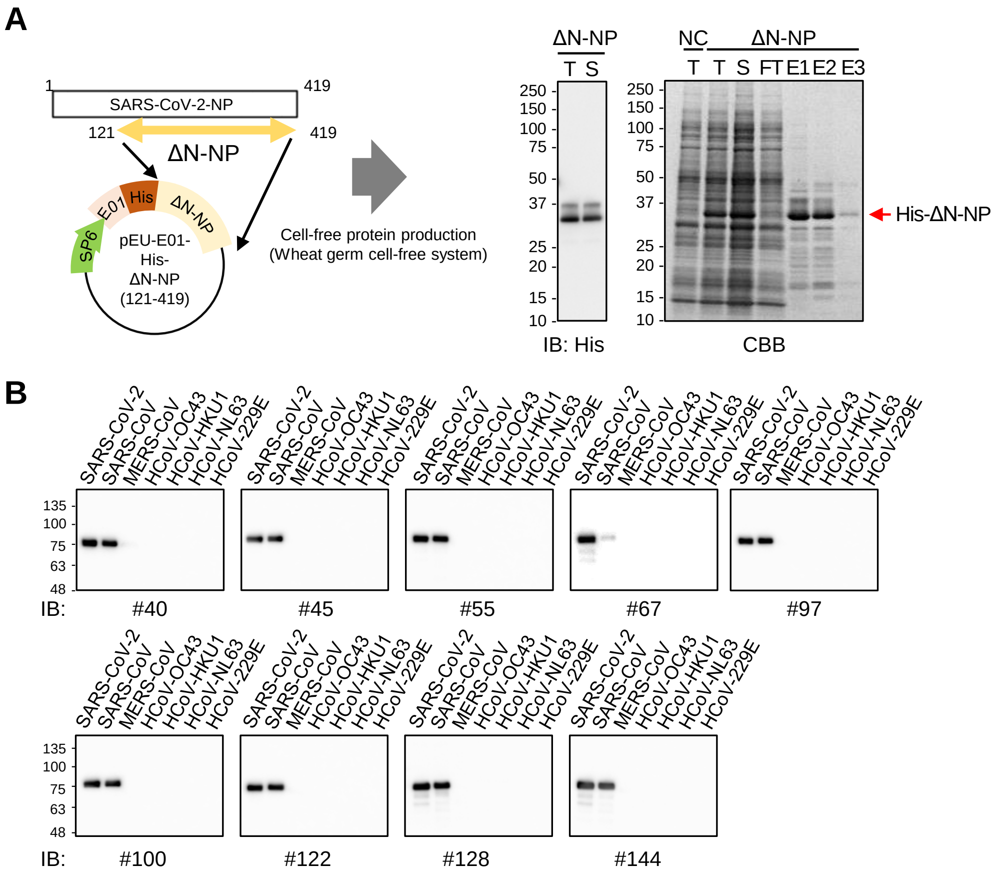

Supplementary Figure 1. (Related to Figure 1) Antigen protein production and specificity screening of developed mAbs.

(A) Schematic representation of antigen protein production. Recombinant Histidine (His)-tagged N-terminally truncated SARS-CoV-2-NP (121-419: ΔN-NP) was produced in a wheat germ cell-free system, and then purified using nickel-chelated Sepharose beads. Each protein fraction was analyzed by SDS-PAGE and visualized by CBB staining. Red arrow indicate the target protein. NC, Negative Control; T, Total fraction; S, Supernatant; P, Precipitate; FT, Flow-Through; E1–3, Elution fractions 1-3. (B) Flag-GST-tagged NPs derived from several human coronaviruses were produced in the wheat germ cell-free system. Reactivity of generated mAbs was validated by immunoblot analysis using the indicated antibodies.

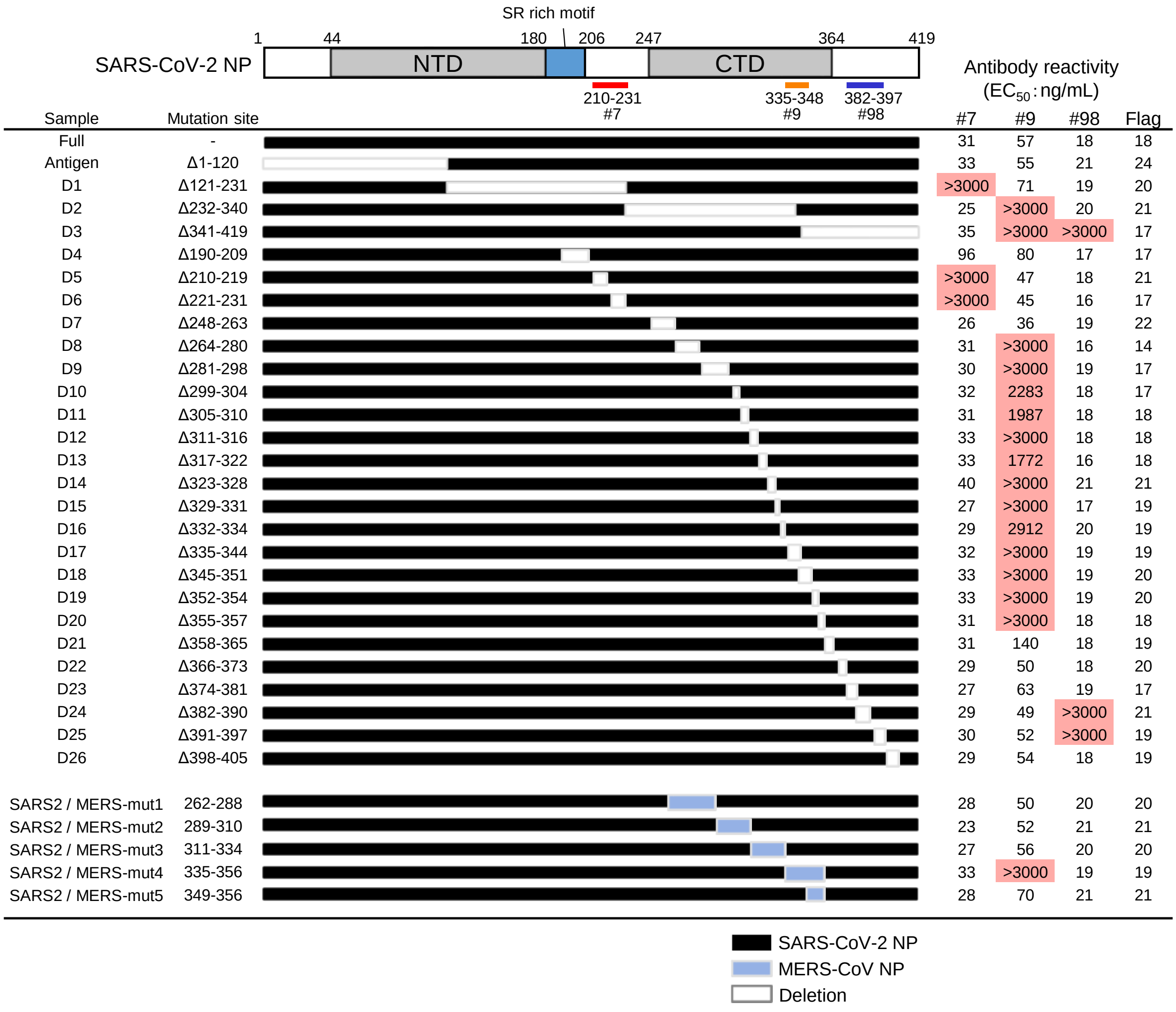

Sample	Mutation site	#7	#9	#98	Flag
Full	-	31	57	18	18
Antigen	Δ1-120	33	55	21	24
D1	Δ121-231	>3000	71	19	20
D2	Δ232-340	25	>3000	20	21
D3	Δ341-419	35	>3000	>3000	17
D4	Δ190-209	96	80	17	17
D5	Δ210-219	>3000	47	18	21
D6	Δ221-231	>3000	45	16	17
D7	Δ248-263	26	36	19	22
D8	Δ264-280	31	>3000	16	14
D9	Δ281-298	30	>3000	19	17
D10	Δ299-304	32	2283	18	17
D11	Δ305-310	31	1987	18	18
D12	Δ311-316	33	>3000	18	18
D13	Δ317-322	33	1772	16	18
D14	Δ323-328	40	>3000	21	21
D15	Δ329-331	27	>3000	17	19
D16	Δ332-334	29	2912	20	19
D17	Δ335-344	32	>3000	19	19
D18	Δ345-351	33	>3000	19	20
D19	Δ352-354	33	>3000	19	20
D20	Δ355-357	31	>3000	18	18
D21	Δ358-365	31	140	18	19
D22	Δ366-373	29	50	18	20
D23	Δ374-381	27	63	19	17
D24	Δ382-390	29	49	>3000	21
D25	Δ391-397	30	52	>3000	19
D26	Δ398-405	29	54	18	19
SARS2 / MERS-mut1	262-288	28	50	20	20
SARS2 / MERS-mut2	289-310	23	52	21	21
SARS2 / MERS-mut3	311-334	27	56	20	20
SARS2 / MERS-mut4	335-356	33	>3000	19	19
SARS2 / MERS-mut5	349-356	28	70	21	21

Supplementary Figure 2. (Related to Figure 1) Epitope mapping of mAbs.

Schematic diagram of SARS-CoV-2-NP domain architecture, mutants, and region of determined epitope of each monoclonal antibody. For epitope mapping, 26 deletion mutants (D1-D26) and 5 substitution mutants (SARS2/MERS-mut1-5) were produced as FLAG-GST-tagged proteins in the wheat germ cell-free system. NTD, N-terminal domain; LKR, flexible linker region; CTD, C-terminal domain. Reactivity of each mAb to deletion mutants was evaluated by indirect-ELISA. EC_{50} was calculated using six different antibody concentrations, and samples with an over 5-fold increase in EC_{50} compared to full-length NP were defined as negative (Red).

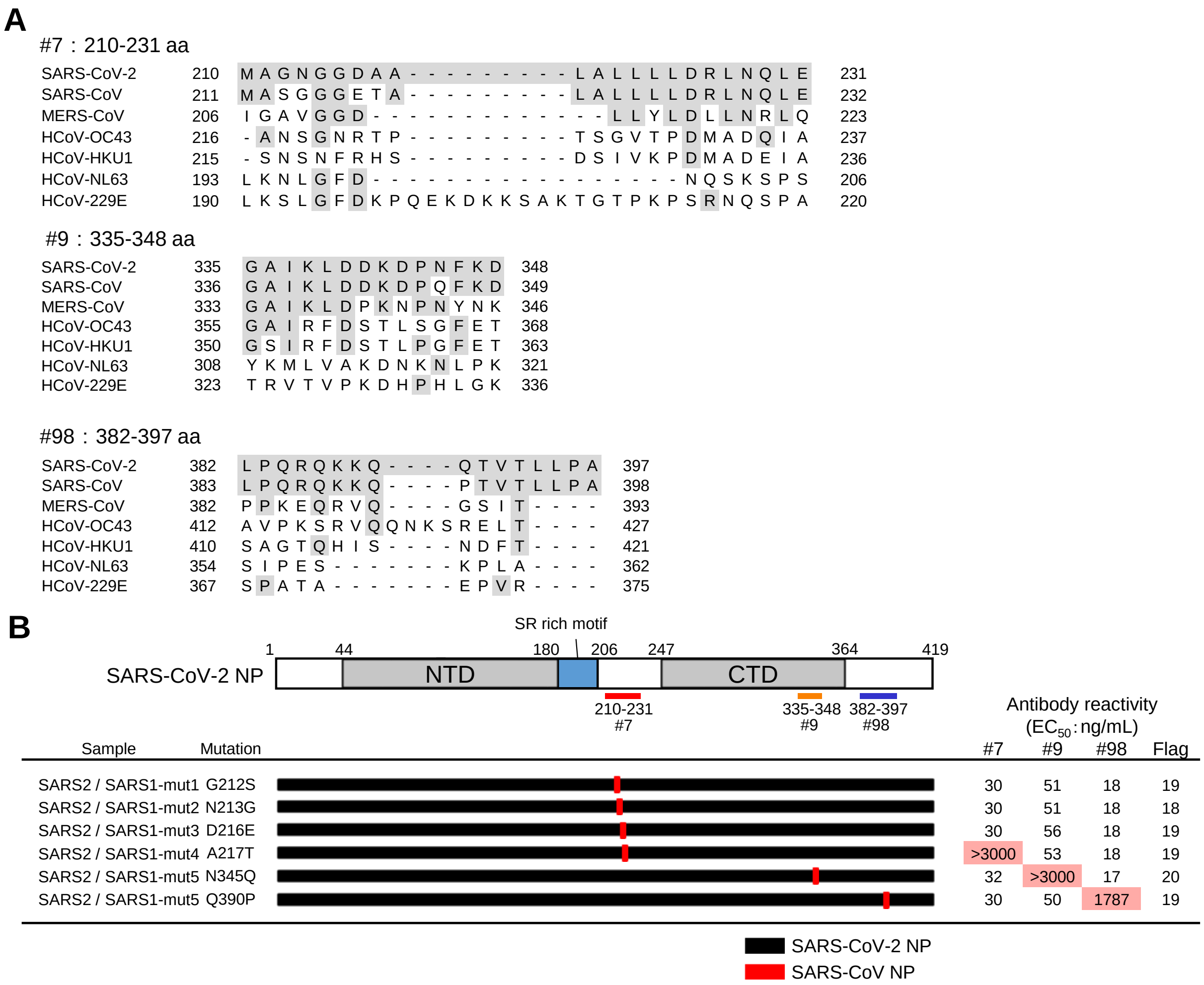

Supplementary Figure 3. (Related to Figure 1) Critical residues for desecting SARS-CoV and SARS-CoV-2 in epitope of developed mAbs.

(A) Multiple sequence alignments of NP of human coronaviruses in the epitope region using MUSCLE software. Gray shaded positions represent conserved residues among the sequences. (B) Determination of critical residues for specific detection of not SARS-CoV but SARS-CoV-2. Five point substitution mutants (SARS2/SARS1-mut1-5) were produced as FLAG-GST-tagged proteins in the wheat germ cell-free system. NTD, N-terminal domain; LKR, flexible linker region; CTD, C-terminal domain. Reactivity of each mAb to deletion mutants was evaluated by indirect-ELISA. EC_{50} was calculated using six different antibody concentrations. The samples with an over 5-fold increase in EC_{50} compared to full-length NP were defined as negative (Red).

A

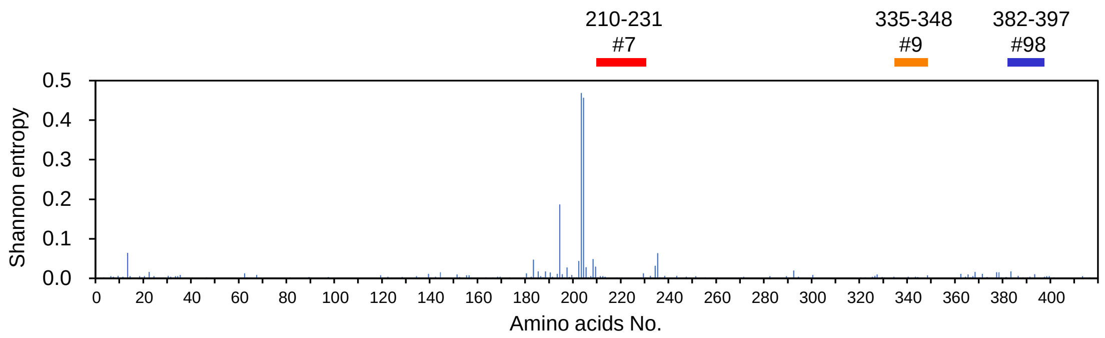

B

#7：210-231 aa

PANGO lineage of SARS-CoV-2		Amino acid sequence of epitope	
A (Prototype)	210	M A G N G G D A A L A L L L L D R L N Q L E	231
B.1.1.7 (501Y.V1)	210	M A G N G G D A A L A L L L L D R L N Q L E	231
B.1.351 (501Y.V2)	210	M A G N G G D A A L A L L L L D R L N Q L E	231
B.1.427	210	M A G N G G D A A L A L L L L D R L N Q L E	231
B.1.429	210	M A G N G G D A A L A L L L L D R L N Q L E	231
B.1.525	210	M A G N G G D A A L A L L L L D R L N Q L E	231
B.1.526	210	M A G N G G D A A L A L L L L D R L N Q L E	231
P.1 (501Y.V3)	210	M A G N G G D A A L A L L L L D R L N Q L E	231
P.2	210	M A G N G G D A A L A L L L L D R L N Q L E	231

#9：335-348 aa

PANGO lineage of SARS-CoV-2		Amino acid sequence of epitope	
A (Prototype)	335	G A I K L D D K D P N F K D	348
B.1.1.7 (501Y.V1)	335	G A I K L D D K D P N F K D	348
B.1.351 (501Y.V2)	335	G A I K L D D K D P N F K D	348
B.1.427	335	G A I K L D D K D P N F K D	348
B.1.429	335	G A I K L D D K D P N F K D	348
B.1.525	335	G A I K L D D K D P N F K D	348
B.1.526	335	G A I K L D D K D P N F K D	348
P.1 (501Y.V3)	335	G A I K L D D K D P N F K D	348
P.2	335	G A I K L D D K D P N F K D	348

#98：382-397 aa

PANGO lineage of SARS-CoV-2		Amino acid sequence of epitope	
A (Prototype)	382	L P Q R Q K K Q Q T V T L L P A	397
B.1.1.7 (501Y.V1)	382	L P Q R Q K K Q Q T V T L L P A	397
B.1.351 (501Y.V2)	382	L P Q R Q K K Q Q T V T L L P A	397
B.1.427	382	L P Q R Q K K Q Q T V T L L P A	397
B.1.429	382	L P Q R Q K K Q Q T V T L L P A	397
B.1.525	382	L P Q R Q K K Q Q T V T L L P A	397
B.1.526	382	L P Q R Q K K Q Q T V T L L P A	397
P.1 (501Y.V3)	382	L P Q R Q K K Q Q T V T L L P A	397
P.2	382	L P Q R Q K K Q Q T V T L L P A	397

Supplementary Figure 4. (Related to Figure 1) Shannon entropy analysis of nucleocapsid proteins and comparison of epitope sequences between prototype and current variant of SARS-CoV-2.

(A) Shannon entropy, as a quantitative measure of variation, was calculated for each amino-acid residue of SARS-CoV-2 NP derived from 8,127 strains. Binding sites of each mAb are indicated in different colors. **(B)** Amino acid sequence in the antigenic epitope of reference strain against each of the currently reported epidemic variant of SARS-CoV-2 [11]. Gray shaded positions represent conserved residues among the sequences.

A

#7 : 210-231 aa

ACCESSION ID	Name of strain	Number of strains	Relative abundance	210	211	212	213	214	215	216	217	218	219	220	221	222	223	224	225	226	227	228	229	230	231
NC_045512	Reference genome	8091	99.56%	M	A	G	N	G	G	D	A	A	L	A	L	L	L	L	D	R	L	N	Q	L	E
MT847220	BGD/BCSIR NILMRC 281/2020	3	0.04%	.	S	.	.	.	.	.	.	.	.	.	.	.	.	.	.	.	.	.	.	.	.
MT706376	USA/WI-UW-249/2020	1	0.01%	I	.	.	.	.	.	.	.	.	.	.	.	.	.	.	.	.	.	.	.	.	.
MT831837	USA/WA-S2367/2020	1	0.01%	V	.	.	.	.	.	.	.	.	.	.	.	.	.	.	.	.	.	.	.	.	.
MT358640	DEU/FFM4/2020	2	0.02%	.	.	.	.	.	.	.	.	.	.	.	.	.	.	.	.	.	.	.	.	F	.
MT873396	USA/MA-MGH-01121/2020	1	0.01%	.	V	.	.	.	.	.	.	.	.	.	.	.	.	.	.	.	.	.	.	.	.
MT628169	USA/CA-CZB-1484/2020	2	0.02%	.	.	.	.	.	.	.	.	V	.	.	.	.	.	.	.	.	.	.	.	.	.
MT472626	USA/IA-CDC-8200/2020	2	0.02%	.	.	.	.	.	.	.	.	.	.	T	.	.	.	.	.	.	.	.	.	.	.
MT601287	BGD/BCSIR NILMRC 75/2020	13	0.16%	.	.	.	.	.	.	.	.	.	.	.	.	.	.	.	.	.	.	.	H	.	.
MT535490	USA/UT-01568/2020	2	0.02%	.	.	V	.	.	.	.	.	.	.	.	.	.	.	.	.	.	.	.	.	.	.
MT614522	USA/WA-QDX-62/2020	2	0.02%	.	.	C	.	.	.	.	.	.	.	.	.	.	.	.	.	.	.	.	.	.	.
MT252734	USA/WA-UW154/2020	4	0.05%	.	.	.	Y	.	.	.	.	.	.	.	.	.	.	.	.	.	.	.	.	.	.
MT451476	AUS/VIC680/2020	2	0.02%	.	.	.	.	.	.	.	.	.	.	.	.	.	.	.	.	.	.	Y	.	.	.
MT806787	USA/IN-QDX-251/2020	1	0.01%	.	.	.	.	C	.	.	.	.	.	.	.	.	.	.	.	.	.	.	.	.	.

#9 : 335-348 aa

ACCESSION ID	Name of strain	Number of strains	Relative abundance	335	336	337	338	339	340	341	342	343	344	345	346	347	348
NC_045512	Reference genome	8107	99.75%	G	A	I	K	L	D	D	K	D	P	N	F	K	D
MT873324	USA/MA-MGH-01405/2020	1	0.01%	.	S	.	.	.	.	.	.	.	.	.	.	.	.
MT706363	USA/WI-UW-235/2020	1	0.01%	.	.	F	.	.	.	.	.	.	.	.	.	.	.
MT326140	USA/WA-UW-1680/2020	2	0.02%	.	.	.	.	.	N	.	.	.	.	.	.	.	.
MT831745	USA/WA-S2426/2020	1	0.01%	.	.	.	.	.	.	.	.	.	.	T	.	.	.
LR877181	SWE/STO-KI/2020	1	0.01%	.	.	.	N	.	.	.	.	.	.	.	.	.	.
MT683418	USA/WA-UW-10138/2020	1	0.01%	.	.	.	.	.	.	.	N	.	.	.	.	.	.
LC529905	JP/TKYE6182/2020	3	0.04%	.	.	.	.	.	.	.	.	.	S	.	.	.	.
MT358737	USA/WA-UW-3994/2020	1	0.01%	.	.	.	.	.	G	.	.	.	.	.	.	.	.
MT509498	IND/GBRC107/2020	4	0.05%	.	.	.	.	.	.	.	.	.	.	.	.	.	Y
MT731292	MAR/RMPS02/2020	2	0.02%	.	.	.	.	.	.	.	.	.	.	.	.	.	H
MT135043	CHN/233/2020	1	0.01%	.	.	.	.	.	.	.	.	V	.	.	.	.	.
MT811401	USA/SEARCH-0834-IPL/2020	2	0.02%	.	.	.	.	.	.	.	.	Y	.	.	.	.	.

#98 : 382-397 aa

ACCESSION ID	Name of strain	Number of strains	Relative abundance	382	383	384	385	386	387	388	389	390	391	392	393	394	395	396	397
NC_045512	Reference genome	8079	99.41%	L	P	Q	R	Q	K	K	Q	Q	T	V	T	L	L	P	A
MT811458	USA/SEARCH-0997-IPL/2020	1	0.01%	.	.	.	.	.	.	R	.	.	.	.	.	.	.	.	.
MT252716	USA/WA-UW180/2020	2	0.02%	.	.	.	.	.	.	.	.	.	.	.	.	.	.	.	S
MT614463	USA/CA-QDX-40/2020	1	0.01%	.	.	.	.	K	.	.	.	.	.	.	.	.	.	.	.
LC571012	JP/DP0654/2020	1	0.01%	.	.	.	.	.	.	.	.	.	.	.	.	.	.	.	V
MT831158	USA/WA-S2249/2020	5	0.06%	.	.	.	.	H	.	.	.	.	.	.	.	.	.	.	.
MT252737	USA/WA-UW146/2020	2	0.02%	.	.	.	.	.	.	.	.	.	I	.	.	.	.	.	.
MT358637	IND/GBRC1/2020	11	0.14%	.	.	.	.	.	.	.	.	.	.	.	I	.	.	.	.
MT757020	USA/FL-BPHL-0351/2020	1	0.01%	.	.	H	.	.	.	.	.	.	.	.	.	.	.	.	.
MT451616	AUS/VIC879/2020	9	0.11%	.	S	.	.	.	.	.	.	.	.	.	.	.	.	.	.
MT646100	USA/MD-HP00086/2020	9	0.11%	.	L	.	.	.	.	.	.	.	.	.	.	.	.	.	.
MT831179	USA/WA-S2105/2020	1	0.01%	S	.	.	.	.	.	.	.	.	.	.	.	.	.	.	.
MT825091	IRN/COVID19-IRVSH1/2020	1	0.01%	.	.	.	.	.	.	.	.	L	.	.	.	.	.	.	.
MT811500	USA/SEARCH-1065-SAN/2020	1	0.01%	.	.	.	.	.	.	I	.	.	.	.	.	.	.	.	.
MT757121	USA/FL-BPHL-0313/2020	1	0.01%	.	.	.	I	.	.	.	.	.	.	.	.	.	.	.	.
MT810952	USA/SEARCH-0240-SAN/2020	1	0.01%	.	.	.	.	.	.	.	.	.	.	.	.	.	.	H	.
MT434815	USA/NY-CDC-SURV0175NYC/2020	1	0.01%	.	.	.	.	.	.	.	.	-	-	-	.	.	.	.	.

B

#7 : 210-231 aa

Name	Mutation site	Antibody reactivity (EC$_{50}$: ng/mL) #7	#9	#98	Flag
Full	-	31	57	18	18
M1	A211S	28	51	17	20
M2	M210I	31	54	18	20
M3	M210V	30	55	18	20
M4	L230F	25	54	19	20
M5	A211V	31	57	18	20
M6	A218V	40	79	19	20
M7	A220T	29	58	18	20
M8	Q229H	27	56	19	21
M9	G212V	36	64	18	16
M10	G212C	153	77	18	17
M11	N213Y	51	74	18	17
M12	N228Y	56	89	17	18
M13	G214C	185	70	17	18

#9 : 335-348 aa

Name	Mutation site	Antibody reactivity (EC$_{50}$: ng/mL) #7	#9	#98	Flag
Full	-	31	57	18	18
M13	A336S	29	57	18	18
M14	I337F	29	62	18	19
M15	D340N	30	56	18	18
M16	N345T	30	63	17	19
M17	K338N	30	57	18	20
M18	K342N	30	52	18	18
M19	P344S	31	2658	16	19
M20	D340G	32	77	17	19
M21	D348Y	32	2348	16	18
M22	D348H	31	1755	17	18
M23	D343V	30	>3000	17	18
M24	D343Y	31	2316	16	17

#98 : 382-397 aa

Name	Mutation site	Antibody reactivity (EC$_{50}$: ng/mL) #7	#9	#98	Flag
Full	-	31	57	18	18
M25	K388R	30	52	16	18
M26	A397S	28	53	16	18
M27	Q386K	29	56	16	18
M28	A397V	29	54	15	19
M29	Q386H	28	54	17	18
M30	T391I	30	52	16	19
M31	T393I	28	50	>3000	19
M32	Q384H	29	56	18	19
M33	P383S	31	63	17	20
M34	P383L	31	58	18	21
M35	L382S	30	60	18	21
M36	Q390L	31	60	98	20
M37	K388I	30	60	19	21
M38	R385I	31	58	18	22
M39	P396H	30	58	15	20
M40	Δ390-392	29	53	>3000	21

Supplementary Figure 5. (Related to Figure 1) Reactivity of developed monoclonal antibodies against recombinant protein from divergent strains.

(A) Identification of amino-acid substitution in the antigenic epitope of each identical strain from 8,127 clinical strains compared with the reference strain. Dots indicate sequence identity relative to the reference strain. Gray shaded

positions represent conserved residues among the sequences. (B) Reactivity of mAbs to recombinant NP from strains possessing amino-acid substitution in the epitope regions. Forty point substitution mutants identified in (A) were produced as FLAG-GST-tagged proteins in the wheat germ cell-free system. Reactivity of each mAb to mutants was evaluated by indirect-ELISA. EC_{50} was calculated using six different antibody concentrations. The samples with an over 5-fold increase in EC_{50} compared to full-length NP were defined as negative (Red).

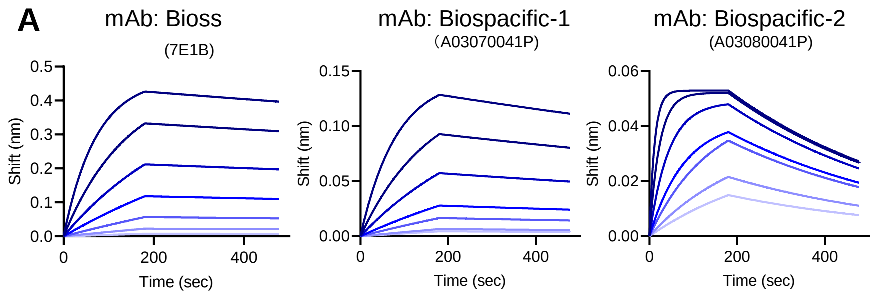

	K_D (M)	k_{on} (1/Ms)	k_{dis} (1/s)
Bioss (7E1B)	9.0E-10	2.7E+05	2.4E-04
Biospacific-1 (A03070041P)	1.2E-09	1.7E+05	2.1E-04
Biospacific-2 (A03080041P)	2.0E-09	1.9E+06	3.6E-03

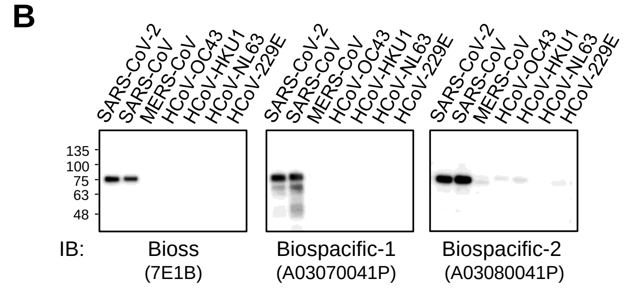

Supplementary Figure 6. (Related to Figure 2) Comparison of commercially available existing mAbs against SARS-CoV-2 NP.

(A) Affinity measurement of commercially available monoclonal antibodies on the octetRED96 instrument. Association and dissociation of each mAbs to full-length NP at various concentrations (50, 25, 12.5, 6.25, 3.13, 1.56 and 0.78 nM) was evaluated using AMC sensor. (B) Specificity of existing mAbs were examined by immunoblot analysis using the indicated recombinant proteins from human coronaviruses.

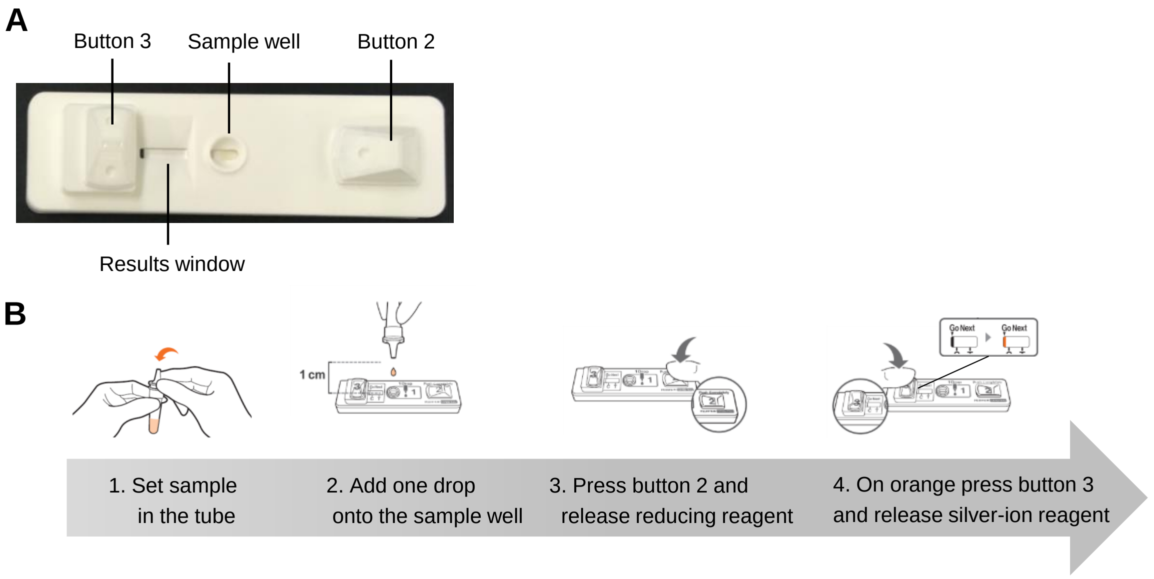

Supplementary Figure 7. (Related to Figure 4) Overview and procedure of LFIA.

(A) Image of LFIA Cartridge. (B) Samples were diluted with an extraction buffer (Tris buffer containing 0.1% non-ionic surfactant) in the tube and one drop of the sample was added onto the sample well of LFIA cartridge. Following this, button 2 was immediately pressed to release a reducing reagent for silver amplification. After the color indicator mark turned orange (about 10 min), button 3 was pressed to release a silver-ion reagent to activate the silver amplification reaction.

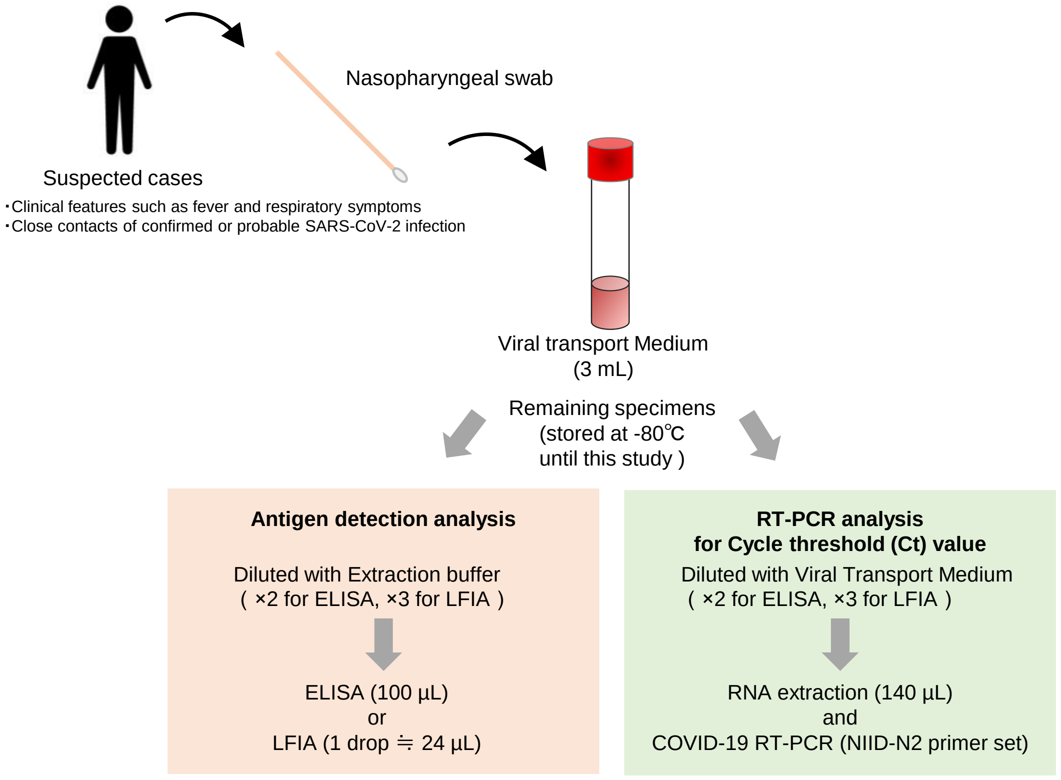

Supplementary Figure 8. (Related to STAR Methods) Evaluation of clinical specimens for antigen detection assay or RT-PCR.

Clinical specimens of nasopharyngeal swabs from cases with suspected SARS-CoV-2 infection were retrieved for this study. Swabs were collected in 3 mL of viral transport medium. RNA extraction and RT-PCR using NIID-N2 set primer were carried out as an administrative inspection. The remaining clinical samples were centrifuged, and the supernatant was used for this study. For antigen detection assays, samples were diluted 2-fold (ELISA) or 3-fold (LFIA) in extraction buffer and 100 µL (ELISA) or 1 drop (LFIA) was subjected to analysis. For RT-PCR analysis to quantify Ct value, samples were diluted to same concentration used in antigen detection assay (2-fold for ELISA, 3-fold for LFIA) with viral transport medium. RNA extraction and RT-PCR analysis were carried out using 140 µL of diluted samples.
